# PanTools v3: functional annotation, classification and phylogenomics

**DOI:** 10.1093/bioinformatics/btac506

**Published:** 2022-07-21

**Authors:** Eef M Jonkheer, Dirk-Jan M van Workum, Siavash Sheikhizadeh Anari, Balázs Brankovics, Jorn R de Haan, Lidija Berke, Theo A J van der Lee, Dick de Ridder, Sandra Smit

**Affiliations:** Bioinformatics Group, Wageningen University, Wageningen 6708PB, The Netherlands; Biointeractions and Plant Health, Wageningen Plant Research, Wageningen 6708PB, The Netherlands; Bioinformatics Group, Wageningen University, Wageningen 6708PB, The Netherlands; Bioinformatics Group, Wageningen University, Wageningen 6708PB, The Netherlands; Biointeractions and Plant Health, Wageningen Plant Research, Wageningen 6708PB, The Netherlands; Genetwister Technologies B.V, Wageningen 6709PA, The Netherlands; Genetwister Technologies B.V, Wageningen 6709PA, The Netherlands; Biointeractions and Plant Health, Wageningen Plant Research, Wageningen 6708PB, The Netherlands; Bioinformatics Group, Wageningen University, Wageningen 6708PB, The Netherlands; Bioinformatics Group, Wageningen University, Wageningen 6708PB, The Netherlands

## Abstract

**Summary:**

The ever-increasing number of sequenced genomes necessitates the development of pangenomic approaches for comparative genomics. Introduced in 2016, PanTools is a platform that allows pangenome construction, homology grouping and pangenomic read mapping. The use of graph database technology makes PanTools versatile, applicable from small viral genomes like SARS-CoV-2 up to large plant or animal genomes like tomato or human. Here, we present our third major update to PanTools that enables the integration of functional annotations and provides both gene-level analyses and phylogenetics.

**Availability and implementation:**

PanTools is implemented in Java 8 and released under the GNU GPLv3 license. Software and documentation are available at https://git.wur.nl/bioinformatics/pantools

**Supplementary information:**

Supplementary data are available at *Bioinformatics* online.

## 1 Introduction

In the field of genomics, attention is shifting toward pangenomics, both in method development and applications in biological research (Bayer *et al.*, 2020). To enable pangenome-based comparative genomics, efficient data structures for sequence compression must be accompanied by methods for data integration and analysis. Where earlier pangenome studies were mostly gene-based, more complex genome-wide representations are currently dominant (The Computational Pan-Genomics Consortium *et al.*, 2018) and there are several methods for pangenome construction (Eizenga *et al.*, 2020). PanTools (Sheikhizadeh Anari *et al.*, 2018) is a representation with a strong focus on generic applicability, data integration and methods for (visual) analytics. Through its distinctive hierarchical graph structure including genomes compressed in a generalized de Bruijn Graph (DBG), structural annotations and homology groups, the heterogeneous pangenome graph can be interrogated using Cypher or PanTools functions. Here, we present PanTools v3, which extends the pangenome graph with new features and provides a new set of command-line tools for powerful comparative genomics analyses. We demonstrate its functionality and performance on five use cases from different taxonomic kingdoms.

## 2 Features

**Fig. 1.**
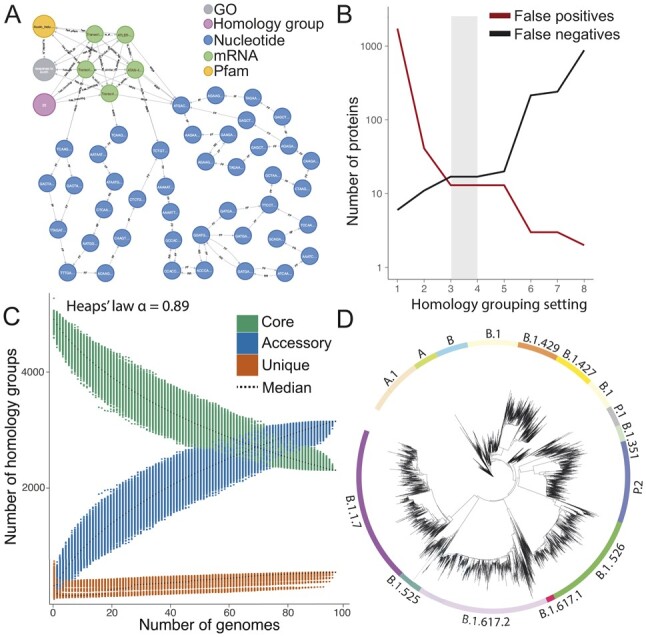
Examples of new features in PanTools v3. (**A**) Part of *Arabidopsis thaliana’s* pangenome graph with five homologous SAUR14 genes sharing two functional annotations. (**B**) Optimal homology grouping obtained in *Pectobacterium* with clustering setting 3 and 4. (**C**) Pangenome growth simulation of *Saccharomyces cerevisiae*. (**D**) *K*-mer distance tree of 10 000 SARS-CoV-2 strains

PanTools v3 offers novel methods for (functional) annotation, gene-level analyses and phylogenetics (all described in more detail in the Supplementary Material):

**Improved annotation:** Next to structural annotations, PanTools can now incorporate the full Gene Ontology (GO) hierarchy (Carbon *et al.*, 2021), Pfam (Mistry *et al.*, 2021) and InterPro (Blum *et al.*, 2021) databases. Functional annotations act as layer in the graph and connect genes sharing a specific function (Fig. 1A). A functionality is available to assess enrichment of connected GO terms. Finally, it is possible to link metadata such as phenotypic information to genetic variability.

**Gene-level analyses:** We extended our pangenomic homology grouping approach with a BUSCO (Waterhouse *et al.*, 2018) benchmark analysis. Assuming that BUSCO genes are single copy, we find optimal settings such that each is placed in a separate homology group with one representative gene per genome (Fig. 1B). Subsequently, a classification method labels genes as core, accessory or unique, and enables copy number variation (CNV) and presence–absence variation (PAV) analysis. CNVs/PAVs can be associated to a phenotype, if available. Sequences in groups can be aligned to identify single-nucleotide polymorphisms (SNPs) or amino-acid changes that can be associated to a phenotype. Pangenome openness (significant gain of novel genes) is determined by iterating over all homology groups, using random genome combinations as proposed by Tettelin *et al.* (2008) (Fig. 1C).

**Phylogenetics:** Comparisons of species or sequences provide meaningful insights when placed in a phylogenetic context. PanTools v3 includes methods to create SNP trees from single-copy genes, consensus trees from multi-copy gene trees, *k*-mer distance and gene distance trees. Two methods, multilocus sequence analysis and Average Nucleotide Identity, were implemented for prokaryotic datasets. Rerooting, clade coloring or altering tree labels is also possible (Fig. 1D).

## 3 Use cases

To demonstrate its new features, we applied PanTools v3 to five datasets from different taxonomic kingdoms: 12 *Drosophila* species, 25 *Arabidopsis thaliana* accessions, 100 *Saccharomyces cerevisiae* strains, 197 strains from the *Pectobacterium* genus and 10 000 severe acute respiratory syndrome coronavirus 2 (SARS-CoV-2) genomes. Construction scalability is demonstrated on *Homo sapiens* and *Solanum lycopersicum*. We created a Snakemake pipeline (Mölder *et al.*, 2021) for reproducibility. A detailed description of the analyses and results is found in the Supplementary Material; here, we highlight a few findings.

- The *A.thaliana* pangenome of 25 accessions is closed, with 75.9–86.3% core genes and only 0.2–0.9% unique genes per accession. Figure 1A shows part of *A.thaliana’*s graph around the SAUR14 gene, with genes clustered into a homology group sharing a GO and Pfam annotation. Furthermore, the DBG (nucleotide nodes) shows different paths due to SNPs and small insertions and deletions. This shows how the integration of sequence, annotation and homology can support pangenomic queries.
- In earlier work, we applied PanTools v3 to a genus-level *Pectobacterium* pangenome (Jonkheer *et al.*, 2021). After finding an optimal homology grouping (Fig. 1B), we could associate 86 homology groups to a virulent phenotype, providing leads for research on protecting plants against this pathogen.
- Genes in the open *S.cerevisiae* pangenome clustered into 39.1% core, 53.1% accessory and 7.8% unique groups (Fig. 1C). Where the original analysis used reference genomes to discover novel genes (Strope *et al.*, 2015), we could efficiently compare all genomes to each other and found genes exclusive to specific populations. These identified groups were enriched with GO terms related to biosynthetic processes.
- A SARS-CoV-2 pangenome was built from a selection of parental lineages and variants currently monitored around the world. As only the reference genome was annotated, a phylogeny was inferred on *k*-mer distances. The resulting classification is highly accurate, although the tree branching (Fig. 1D) does not reflect the actual phylogeny well. Our *k*-mer method produced phylogenies highly similar to the alignment-based method in <1% of the runtime.

Overall, the results demonstrate that PanTools is applicable to genome collections of different sizes and complexity. Construction of the pangenome graph currently scales to thousands of bacteria, hundreds of fungi and depending on genome complexity, dozens of animal and plant genomes. PanTools’ alignment-free representation is not limited to within-species analyses but can work at the genus or family level.

## 4 Conclusion

PanTools v3 enables large-scale comparative genomics in pangenomes by including (functional) annotations and offering methods to analyze genome content, organization and phylogeny. PanTools is easily installed and comes with an extensive manual. We successfully used the platform to analyze genetic diversity in the *Pectobacterium* genus and demonstrated its broad applicability here in four additional use cases. With increasing interest in pangenomes, PanTools has the potential to be used in many comparative genomics projects.

## Funding

This research was funded by the Dutch Ministry of Economic Affairs in the Topsector Program ‘Horticulture and Starting Materials’ (project number: TU 16022) and its partners (NAK, Naktuinbouw and BKD).

*Conflict of Interest*: none declared.

## Supplementary Material

btac506_Supplementary_Data

## Data Availability

The data underlying this article are available in the 4TU.ResearchData repository, at https://doi.org/10.4121/19874485.
