# Supplementary material for: PanTools v3: functional annotation, classification and phylogenomics
Source: Bioinformatics. 2022 Jul 21;38(18):4403–5. doi: 10.1093/bioinformatics/btac506 (PMC9477522; doi:10.1093/bioinformatics/btac506)
Supplement: btac506_Supplementary_Data [file btac506_supplementary_data.zip › btac506_Supplementary_Data/Pantools_v3_supplement.pdf]

PanTools v3: Supplementary information

Supporting material for the publication “PanTools v3: functional annotation, classification, and phylogenomics”

1 Introduction

PanTools is a platform for comparative analyses of large numbers of genomes. Version 3 brings improved pangenome annotations, functions to analyze the gene repertoire, and methods to construct phylogenetic trees. This document provides detailed descriptions and examples of the novel functionalities. In earlier work, the software was used for a genus-level analysis of 197 *Pectobacterium* genomes [1]. Here, we demonstrate the scalability and applicability from different taxonomic kingdoms using publicly available genome data from *Homo sapiens*, *Solanum lycopersicum*, *Drosophila* [2], *Arabidopsis thaliana* [3][4], *Saccharomyces cerevisiae* [5] and SARS-CoV-2. A Snakemake pipeline was created to reproduce the pangenome analyses on the seven datasets.

1.1 PanTools data structure

The PanTools data structure is a combination of a sequence representation and feature layers that enable efficient traversal (Fig. 1). The data is stored in a Neo4j (v3.5.30) graph database, with nodes and directed edges (relationships) carrying name-value pairs, so-called properties. Nodes can have multiple labels, whereas edges have only one label. At the basis of PanTools is an indexed and compressed De Bruijn Graph (DBG) with several features for high efficiency [6]. Each node in the DBG corresponds to a unique k-mer, each edge represents an overlap of $k-1$ between two k-mer sequences. The genomic positions of k-mers are stored on the edges. To reduce the number of nodes in the graph, non-branching paths are collapsed into a single node. Genome annotations (genes, mRNAs, CDS, tRNA) are stored in a layer of nodes linked with edges to their start and stop positions in the DBG. Homologous (protein coding) genes are connected through homology groups, which connect genes from different genomes [7]. In version 3 we introduced functional annotation nodes connecting genes sharing a particular function.

1.2 Graph queries using Cypher

Cypher is Neo4j’s graph query language enabling retrieval of data from the graph. The language uses an ASCII-art type of syntax for matching node and relationship patterns. Cypher allows users to ask very specific questions to the database, for example: "return genes of a length between 200 and 500 bp that have an annotated signal peptide and a certain GO identifier". In our online manual we introduce users to the language and provide a set of example queries. Cypher queries are limited however, because traversal of a path in a genome requires using the genomic coordinates stored in the edge attributes. For such, more complex, queries dedicated PanTools functions should be used.

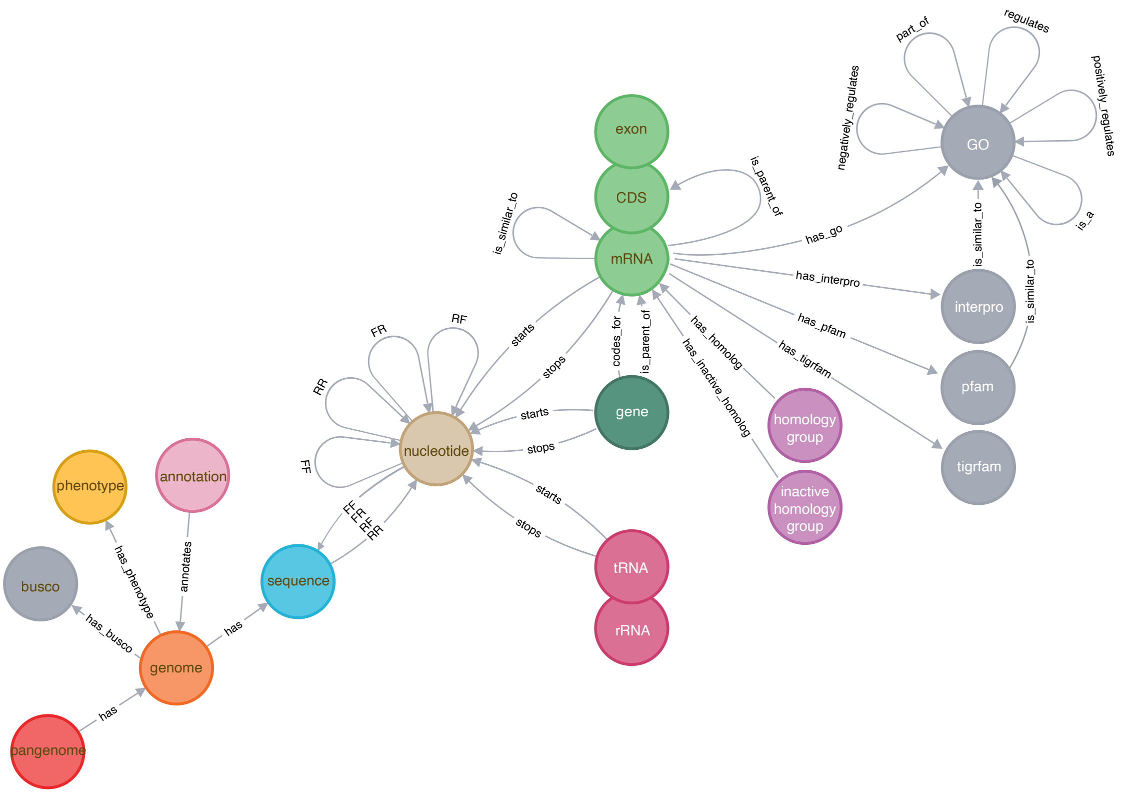

Figure 1: The PanTools graph database scheme visualized in the Neo4j browser. To prevent overlapping edges in this visualization, similar annotation nodes are stacked and redundant 'starts' and 'stops' relationships are removed.

2 Features

PanTools v3 offers novel functionalities in three categories: improved pangenome annotation, extensive gene-level analyses, and phylogenetic analyses. In this section we briefly describe these new features. A complete explanation on how to use the (new) PanTools functionalities is available at https://git.wur.nl/bioinformatics/pantools. The manual gives step-by-step instructions, lists command line arguments, illustrates example input files and describes the output files.

2.1 Improved pangenome annotation

2.1.1 Functional annotations

PanTools v3 provides easy ways to incorporate the output from a variety of annotation pipelines and the content of various functional databases: GO [8], Pfam [9], InterPro [10], TIGRFAM [11], COG [12], Phobius [13] and SignalP [14]. From GFF files produced by InterProScan [15], we extract and incorporate the mentioned annotation types except COG. Output from the standalone Phobius (1.01) and SignalP (4.1 and 5.0) can be included as alternative to InterProScan. The output of eggNOG-mapper [16] is parsed to identify COG gene functions as well as GO terms.

Finally, PanTools can read custom-made annotation files that consist of two tab or comma-separated columns. The first column should contain a gene identifier, the second an identifier from one of four functional annotation databases: GO, Pfam, InterPro or TIGRFAM. This allows users to use annotation software of their own preference, only requiring a minimal effort to convert output to this two-column format.

As functional databases receive constant updates, we frequently update the database versions in the PanTools Git repository. Due to its large size, InterPro is downloaded when any functional annotation functionality is initialized for the first time. When annotation software uses different database versions from those integrated into the pangenome, annotations with mismatching identifiers cannot be be incorporated. Users can manually update the database files, following instructions in the online manual.

Annotations enable the biological interpretation of genes, but also serve to link genes. The first time a user adds functions to a pangenome, the databases are fully integrated. Each entry of the GO, InterPro, Pfam and TIGRFAM databases is stored in a separate node and genes with a predicted function are assigned to that node through an edge. Pfam and InterPro domains associated with a GO term are also connected. GO functions are structured in a directed acyclic graph (DAG) where a node can have multiple (more specific) child nodes and more than one (less specific) parent nodes. Edges between GO nodes in the hierarchy can have different type of labels: *is a*, *part of*, *has part*, *regulates*, *negatively regulates* and *positively regulates*. For COG annotations, we store the name, the functional category and name of the metabolic pathway directly in the mRNA nodes. For Phobius and SignalP, the transmembrane domain and signal peptide information is also stored in mRNA nodes.

We implemented methods to create clear summaries of functions connected to an individual gene, to genes in a homology group or to all genes in the pangenome. PanTools can identify over- or under-represented GO terms in sets of genes using a hypergeometric test. The user can select a significance threshold (default 0.05) and the multiple testing correction procedure, Bonferroni [17] or Benjamini-Hochberg [18]. GO hierarchies can be visualized by PanTools, showing the GO terms, their relationships and statistical significance (Fig. 2). Beside these functionalities using the integrated functions in the database, the highly interconnected graph structure of the pangenome can be utilized to annotate genomes with no or limited functional annotations. As homology group nodes connect genes from different genomes, only a single annotated gene node is required for linking a function to other homologous genes. Therefore, it might be beneficial to include the genome of a related and well-annotated species to enhance the functional annotation analysis. For example, *A. thaliana*, as the most studied plant in the world, will contribute to the functional analysis within its own *Brassicaceae* family, possibly even at a higher taxonomic rank. High-quality annotations, as typically found in model organisms, are characterized by a high BUSCO score and gene models that are supported by expression and experimental evidence, functional annotations and homology.

2.1.2 Phenotype incorporation

To improve understanding of the genotype-phenotype relationship through comparative (pan)genomics, phenotype information can be incorporated in the pangenome data structure. PanTools methods can use this to find similarities for a group of genomes sharing a phenotype or to identify variation between different phenotypes. As phenotypic data can be heterogeneous, text (string), numeric (integer, double), or true/false (boolean) values are allowed. Genomes are only considered to share the same phenotype when values are identical. Optionally, numerical values can be grouped into equally sized bins set by the user (default of 3 bins). Bins of unequal

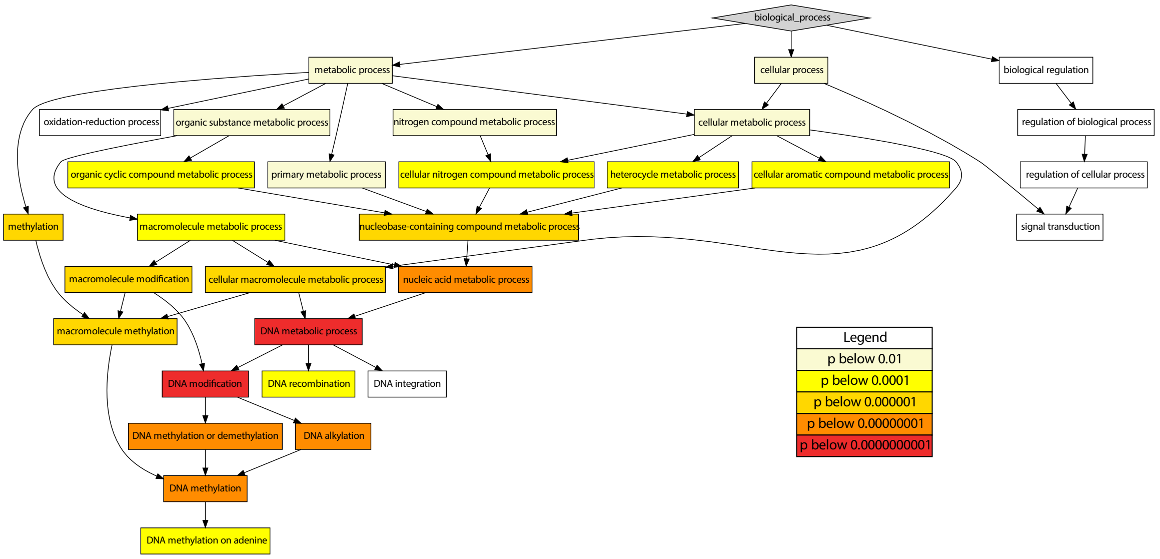

Figure 2: Visualization of biological process GO annotations by dot [19]. Box colors indicate p-value ranges. GO terms were retrieved from virulence associated genes in the *Pectobacterium* pangenome (section 3.4).

distribution must be prepared manually.

After incorporation, individual phenotypes can be used by the classification and sequence alignment methods (described later on), e.g. to find all virulent-specific genes, to report single nucleotide polymorphisms (SNPs) shared by tomatoes with purple fruits, or to identify functional annotations specific to short winged flies. Moreover, phenotype values can be integrated in phylogeny leaves allowing phylogenetic tree annotation.

2.2 Gene-level analyses

2.2.1 Optimal protein clustering

Clustering proteins into groups of homologs (orthologs and paralogs) is the starting point for many genomic comparisons. Finding the most suitable settings for homology grouping is challenging and depends on evolutionary distance of the genomes, which is not always known *a priori*. PanTools v3 adds a method to optimize settings, performing homology grouping on a maximum of 8 different relaxation settings, from most strict (d1) to most relaxed (d8) and evaluating performance using BUSCO genes (F_1 score, see below). If specified, the search for a grouping can automatically be stopped after performance of the grouping decreases compared to the previous setting. All groupings except the optimal one are then inactivated but kept in the database. Therefore, the user can alternate between groupings to adjust the strictness of the clustering without having to re-run homology grouping.

Grouping performance is evaluated based on BUSCO genes [20]. We assume that a BUSCO gene is truly single-copy and a perfect grouping would place each BUSCO gene in a separate homology group with one representative protein per genome. Only complete and non-duplicated BUSCO genes present in all genomes are used to measure the performance; these are identified by running BUSCO (version 3, 4, or 5) with any odb9 or odb10 dataset against the protein sequences in the

pangenome database. The effectiveness of this benchmark analysis relies on the specificity of the available BUSCO lineages. To illustrate, a family-level *'Solanaceae'* set (5950 genes) can be selected for a genus-level *Solanum* pangenome (containing tomato for instance), whereas for the *Lactuca* genus (lettuce) the less specific 'eudicots' (2326 genes) is the most suitable single-copy gene set.

Performance is evaluated for each BUSCO gene (Fig. 3), as follows: when all proteins corresponding to the BUSCO gene are grouped together, these are considered true positives (*tp*) and additional non-BUSCO genes in the group are counted as false positives (*fp*). When proteins of a BUSCO gene are found in multiple groups, the highest number of proteins present in one group are considered *tp*. Again, other proteins in this group are considered *fp*. Proteins corresponding to the BUSCO gene in other groups are counted as false negatives (*fn*). The sums of *tp*s, *fp*s and *fn*s are defined as TP, FP and FN, respectively. From these scores recall, precision and F_1 score measures are calculated as follows:

$$Recall = \frac{TP}{TP + FN} \tag{1}$$

$$Precision = \frac{TP}{TP + FP} \tag{2}$$

$$F_1\ score = \frac{2 * Precision * Recall}{Precision + Recall} \tag{3}$$

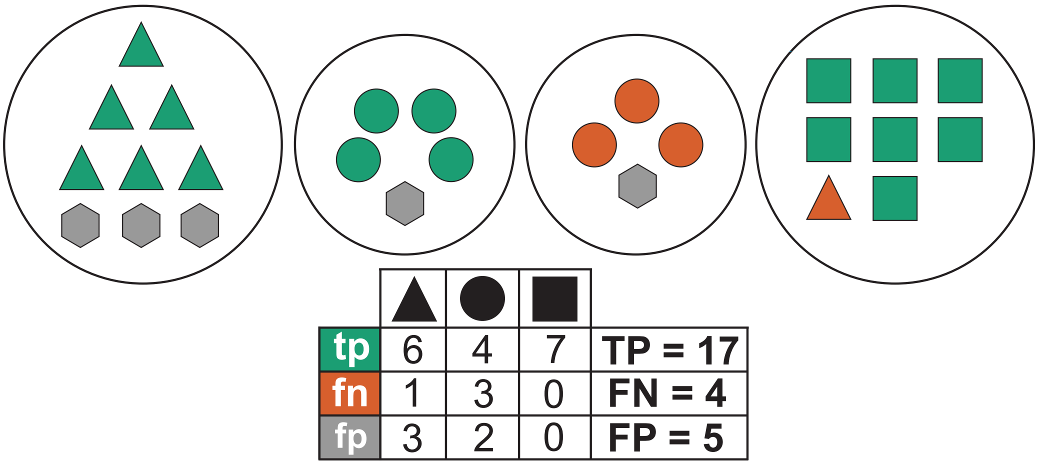

Figure 3: Illustration of performance evaluation for four homology groups. BUSCO genes are represented as triangles, circles and squares. Green shapes are true positives (tp), red shapes false negatives (fn). All non-BUSCO genes (grey hexagons) are counted as false positives (fp).

2.2.2 Classification

Pangenome gene content is typically described as core (genes present in all genomes), accessory (present in some genomes) and unique (present in one genome) [21]. For a set of genomes sharing a phenotype, we include three additional categories: shared (present in all genomes with that phenotype and possibly in one or more additional genomes), exclusive (only present in genomes with a certain phenotype) and specific (shared by and exclusive to genomes with a certain

phenotype) (Fig. 4). Gene duplications do not affect this classification; for instance, when a single genome has additional gene copies where other genomes do not, we still consider these copies to be core. As core and unique categories are highly influenced by the quality of the genomes and annotations in the dataset, thresholds can be specified to allow the identification of so-called softcore (>95% occurrence) or shell genes (<1%).

**Homology group K-mer Function**	Phenotype 1					Phenotype 2			Phenotype 3				**Definition**
	G1	G2	G3	G4	G5	G6	G7	G8	G9	G10	G11	G12	
1	1	3	1	1	2	1	1	1	1	1	1	1	Core
2	1	0	1	1	1	1	1	0	0	0	1	1	Accessory
3	0	0	1	0	0	0	0	0	0	0	0	0	Unique
4	1	1	1	1	2	0	1	2	2	1	0	0	Phenotype shared
5	1	0	1	0	1	0	0	0	0	0	0	0	Phenotype exclusive
6	1	1	1	2	1	0	0	0	0	0	0	0	Phenotype specific

Figure 4: Definition of the different categories that can be assigned to genes, k-mers and functional annotations.

We implemented the classification method for genes and two additional categories, k-mer sequences and functional annotations, purely based on presence/absence (Fig. 4). Classification of gene content enables copy number variation (CNV) and presence-absence variation (PAV) analyses. The classification of k-mers can help find strain- and phenotype-specific barcodes and counts k-mers to estimate similarity between genomes (see section 2.3.4). By classifying functional annotations, users can retrieve (non)shared functions for a collection of genomes. All three methods report the number and percentage of classified elements for the pangenome and individual genomes. Output files for each per category are generated holding node identifiers that are usable for database queries. In addition, a Fisher's exact test can be applied to associate homology groups with phenotypes. The gene classification function produces an additional set of files specific for gene CNV analysis. For instance, single-copy orthologs are reported in addition to core genes and groups are reported where one or more genomes have an increased number of gene copies compared to at least one other genome. When a phenotype is included, homology groups can be found of which all genomes have at least one extra copy compared to all genomes of another phenotype.

2.2.3 Co-localized genes

The genomic location of genes plays a role in shaping how an organism's traits evolve. Several studies have shown examples of co-expressed and co-localized genes, implicating their involvement in a similar process. From a set of homology groups this function identifies gene clusters of neighbouring genes. Gene neighbours are considered to be directly co-localized without intervening genes and be within a distance of 1 Mb. The user can set an allowed number of non co-localized genes per cluster and change the maximum allowed distance. These strict rules result in the identification of small gene clusters unlike synteny methods constructing large collinear blocks overlapping entire chromosomes using gene anchors.

First, this PanTools function retrieves the gene order in genomes, checking whether genes in the selected homology groups are co-localized. The detected gene clusters are reported per genome. When multiple genomes are included, cluster compositions are compared to identify shared (sub)clusters between genomes. Genes of a (sub)cluster are allowed to be in opposite orientation.

From the pairwise comparisons, conserved clusters between two genomes are reported. If certain (sub)clusters are present in all genomes of the current analysis, they are marked as core cluster. Whereas a core cluster considers all genomes (in the analysis), including a phenotype allows users to find shared (sub)clusters for a specific set of genomes.

2.2.4 Pangenome structure estimation

The structure of a pangenome can be described as open or closed [21]. A closed structure suggests the gene pool is saturated, an open structure implies a significant number of novel genes are discovered in each genome added to the pangenome. The growth and openness of the pangenome are determined by iterating over all homology groups with random genome combinations, according to the models proposed by Tettelin *et al.* (2008) [22]. Each iteration starts with three random genomes from which core, accessory and unique homology groups are identified. Subsequently, random genomes are added and groups are reclassified until all genomes have been included. The contribution of each added genome is calculated as the increase in core, accessory, and unique homology groups. A minimum of 1,000 iterations is required, but at least 10,000 is recommended.

Heaps' law [22] function is fitted to the distribution of the number of new genes observed when increasing the pangenome:

$$n = kN^{-\alpha} \tag{4}$$

where n is the newly discovered genes, N is the total number of genomes, and k and α are the fitted parameters. The pangenome can be considered open when $\alpha < 1$ and closed if $\alpha \geq 1$.

2.2.5 Sequence alignments

We incorporated MAFFT [23] in the PanTools conda environment to enable multiple sequence alignments in the pangenome. The user can align sequences using four different approaches: per single homology group (1), for a combination of groups (2), sequences sharing a functional domain (3) or a manual selection of genomic regions (4). When aligning genes in homology groups, both nucleotide and amino acid sequences are aligned. The longest start and end gaps in the protein alignment are used to trim the input sequences; thereafter, trimmed sequences are aligned a second time (by default, but this can be disabled). For the alignment of genomic regions, trimming and re-alignment is not possible. The sensitivity of MAFFT is controlled by including a BLOSUM (45, 62 or 80) argument, remaining parameters are default. The alignments run in parallel when multiple threads are assigned to PanTools.

Alignments are stored in both CLUSTAL and FASTA format, from which a range of statistics and additional files are created. FastTree 2.1.10 [24] is applied to the alignments using default parameters in order to create ML gene trees. Pairwise identity and similarity scores are reported for all sequence pairs; a BLOSUM (45, 62 or 80) matrix is used to calculate protein similarity. The alignments are read to identify conserved, variable and parsimony-informative sites. When a phenotype is included in the analysis, variable positions in alignments are used to identify phenotype-specific SNPs or amino acid substitutions. In addition, the distribution of phenotypes for each variant is reported.

2.3 Phylogeny

A phylogeny describes the evolutionary history and relationships among organisms and is fundamental for answering many biological questions. We implemented six commonly-used phylogenetic strategies in PanTools. Distance-based approaches use Neighbour Joining (NJ) and

are highly effective and easily scalable to large datasets, at the cost of lower accuracy. The Maximum Likelihood (ML) method is powerful, but not efficient enough for pangenomes with hundreds of genomes. All six functions produce Newick-formatted tree files that contain the species tree topology, branch lengths and branch support values (only for ML methods).

2.3.1 Core phylogeny

Core phylogenies estimate the taxonomic relationships in a set of genomes using genetic variation found in core genes. With PanTools we infer a core phylogeny on all parsimony-informative single-nucleotide polymorphisms (SNPs) or amino-acid substitutions of single-copy orthologous genes. First, nucleotide or protein sequences of single-copy groups are aligned with MAFFT (section 2.2.5). Subsequently, parsimony-informative positions are retrieved and concatenated into a single contiguous sequence per genome. These sequences serve as input for IQ-tree [25] to infer a ML tree with default parameters and a minimum of 1,000 bootstrap replicates. A command is generated to run IQ-tree with ModelFinder, default parameters and 1,000 bootstrap replicates. PanTools generates the required IQ-tree command but does not execute it. In this way the arguments can be updated by the user. For example, increasing the number of bootstrap replicates or selecting a specific substitution model. The two main output files from IQ-tree are the consensus tree and split graph tree. A consensus tree is a regular phylogenetic tree with bootstrap support values on its branches. Depending on a conflicting phylogenetic signal, the split graph tree is more like a network and visualizes all splits supported by bootstrap trees.

2.3.2 MLSA

A multi-locus sequence analysis (MLSA) is a traditional method widely used for prokaryotic strain classification that only considers variation in a limited set of (housekeeping) genes [26]. MLSA is fast and does not require full genome sequences, but generally provides a weak phylogenetic signal compared to the core phylogeny. We implemented a function to perform a MLSA. In the initial step, gene candidates are identified and filtered based on being present as single-copy in all (selected) genomes. When a gene is present in all genomes but not single-copy, its sequences are aligned and a gene tree is created. The user can then manually remove any outlying sequences from the analysis. If outlier removal results in a gene becoming single-copy, the sequences can be used in MLSA. In addition, the user is warned when a selected gene is clustered into multiple homology groups, as this suggests a high dissimilarity between sequences. After gene selection, sequences are aligned and trimmed using the method described in section 2.2.5. Alignment output is combined into a single contiguous sequence per genome from which a ML phylogeny is inferred with IQ-tree using default settings [25]. IQ-tree produces a consensus tree and a split graph tree.

2.3.3 Consensus tree

Although gene trees generally do not agree with species trees, many methods are developed that estimate phylogeny by combining gene trees into a consensus tree [27]. Most tools require single-copy genes, but because genes duplicate, are lost or incorrectly annotated, the number of single-copy groups can be insufficient for an accurate species tree. ASTRAL-Pro [28] was designed to handle multi-copy gene trees and was therefore incorporated into PanTools. To create a consensus tree, we first align protein sequences in all homology groups, as described in section 2.2.5 section. Thereafter, one (ML) gene tree is inferred for each homology group using FastTree 2.1.10 [24] with default settings. Both the alignment and tree inference are parallelized when multiple threads are assigned

to PanTools. All gene trees are combined into a single file which is supplied to ASTRAL-Pro, with default parameters.

2.3.4 k-mer distance tree

With increasingly large datasets, calculating phylogenetic distances is shifting to alignment-free methods. We introduced a k-mer counting method in PanTools that counts the number of (shared) k-mer sequences in genomes in the DBG. Because the graph is compressed, k-mers from non-branching paths are collapsed into a longer sequence and represented by a single node. The actual number of k-mers in a node is determined by subtracting $k-1$ from the compressed k-mer length, where k is the original k-mer length used to build the pangenome. Nodes where the sequence contains degenerate bases (not A, T, C, G) are excluded. The user can select three types of distance to infer a phylogenetic tree from k-mer counts. Two are Jaccard distances, the first only considering unique elements whereas the second considers all elements:

$$D_J(A,B) = 1 - J(A,B) = \frac{|A \cup B| - |A \cap B|}{|A \cup B|} \tag{5}$$

$$D_J(A,B) = 1 - J(A,B) = \frac{|A \uplus B| - |A \cap B|}{|A \uplus B|} \tag{6}$$

Third, the MASH distance as described by Ondov *et al.* in 2016 [29]. Distances are placed in a matrix from which a NJ tree is inferred using the ape R package v5.0 [30].

2.3.5 Gene-distance tree

A distance based on gene (family) absence/presence is a simple yet effective basis to classify sequences or genomes. Homology groups are used to count the number of genes shared between two genomes. The user can select one of two Jaccard distances (see 2.3.4) to infer a phylogenetic tree: one distance ignores additional copies of genes whereas the other distance includes all gene copies. Distances are placed in a matrix from which a NJ tree is inferred using the ape R package v5.0 [30].

2.3.6 ANI-distance tree

The Average Nucleotide Identity (ANI) is a pairwise metric, commonly used for strain identification and determination of species boundaries in prokaryotes. The original metric is based on the mean percent identity of reciprocal best BLAST hits [31]. Novel methods that approximate ANI show high correlation with the traditional method, but come with significant runtime improvements. Therefore we estimate ANI scores by FastANI (v1.32) [32] or MASH (v2.2.2) [29]. To create a phylogeny, ANI scores are transformed as 1-(ANI/100) to a distance in the range 0–1. Distances are placed in a matrix from which a NJ tree is inferred using the ape R package v5.0 [30].

2.3.7 Tree editing

After a phylogenetic tree is created it can easily be updated by (re)rooting (i), changing the text of the tree labels (ii) and coloring labels according to a phenotype (iii). A tree can be unrooted (Fig. 5A) rooted (Fig. 5B) or rerooted on the external nodes, for which we use ape v5.0 [30]. Tree label text can easily be annotated using phenotype information stored in the pangenome. Optionally, genome numbers can be included (Fig. 5B) or excluded (Fig. 5C) from the labels. The third functionality generates templates to color tree labels by phenotype in iTOL v5 [33], an intuitive web-based tool for

the visualization of phylogenetic trees. It supports several annotations, such as coloring of branches, labels and clades. The required input file to allow phylogeny coloring requires manual work or custom scripting which is no longer required due to the PanTools templates.

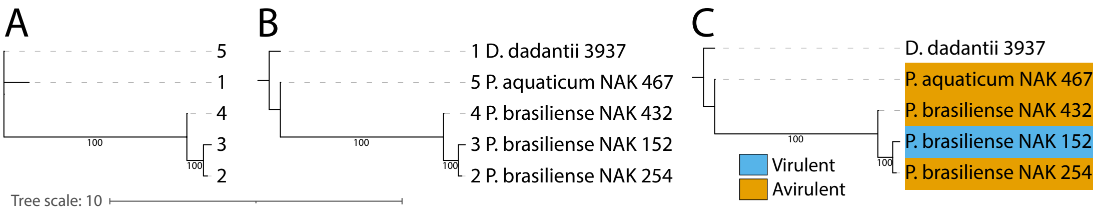

Figure 5: Example of editing a phylogenetic tree with one *Dickeya* and four *Pectobacterium* strains. (A) An unrooted core SNP tree inferred by IQ-tree only contains genome numbers when no phenotype was included. (B) The tree is rooted using the *Dickeya* genome and strain information is incorporated onto the labels. (C) Tree labels are colored according to the virulence phenotype and genome numbers are removed.

3 Use cases

The following use cases demonstrate the applicability and scalability of the new functionalities to different (super)kingdoms of life. The genome and annotations were obtained from public repositories (see Additional file 1, Table S1-S5) and functionally annotated with InterProScan (5.52-86) [15] and eggNOG-mapper v2 [16]. All analyses were performed on a Linux server (Ubuntu 18.04) with an Intel(R) Xeon(R) CPU E5-2690 v4 @ 2.60GHz, with 56 CPUs and 252 GB RAM. The Java virtual machine (JVM) controls the memory usage of PanTools and is created with a minimum of 20 GB of space with a maximum heap size of 80 GB. Runtimes for the construction and analyses are shown in Table 1.

Table 1: Total wall-clock time (hh:mm:ss) of PanTools functionalities.

**Function**	**Threads**	**12** *Drosophila*	**25** *A. thaliana*	**100** *S. cerevisiae*	**197** *Pectobacterium*	**10,000** **SARS-CoV-2**
build_pangenome	1	11:58:01	06:14:27	02:14:32	08:53:02	37:09:06
add_annotations	1	00:25:17	00:12:21	00:03:43	00:23:39	00:00:10
optimal_grouping	24	06:27:53	04:45:55	05:20:29	12:19:20	-
add_functions - InterProScan	1	00:02:57	00:04:27	00:03:32	00:08:38	-
add_functions - eggNOG	1	00:11:50	00:05:31	00:02:44	00:10:14	-
pangenome_structure	24	00:00:19	00:00:41	00:01:08	00:1:54	-
gene_classification	1	00:00:24	00:00:08	00:01:05	00:00:45	-
k-mer_classification	1	00:19:42	00:09:29	00:33:00	00:11:07	00:58:50
function_classification	24	00:00:05	00:00:04	00:00:05	00:00:12	
Alignments (Core SNP & Consensus tree)	24	01:15:19	06:26:23	06:19:07	04:08:25	-
Core SNP tree - IQ-tree	24	00:23:50	00:04:49	00:08:02	38:55:43	-
Consensus tree - ASTRAL-Pro	1	00:00:08	00:09:44	00:38:08	01:43:53	-
ANI distance tree	24	-	-	-	01:49:03	-
MLSA	24	-	-	-	00:12:53	-

3.1 *Drospohila*

The *Drosophila* genus (NCBI:txid32281) is a model organism in different fields of biology. Research has yielded a reasonable number of fully sequenced genomes, making it a suitable species for an animal pangenome. Curated genome and annotation data for 12 *Drosophila* species were obtained from Flybase [2]: *D. ananassae*, *D. erecta*, *D. grimshawi*, *D. melanogaster*, *D. mojavensis*, *D. persimilis*, *D. pseudoobscura*, *D. sechellia*, *D. simulans*, *D. virilis*, *D. willistoni* and *D. yakuba* (Additional file 1, Table 1). The genome size varied strongly between species, the largest genome of 235 Mb *D. willistoni* being almost twice the size of the 125 Mb *D. simulans* genome. The GC content ranged between 37.3-44.9%. Interestingly, the number of genes varies from 14,845 to 18,240, but does not correlate with genome size. For example, with 15,134 genes *D. willistoni* had substantially less genes than 18,240 *D. simulans*. The order-level diptera odb10 BUSCO set was used to obtain the optimal grouping.

The d6 grouping ($\geq$ 45% protein similarity) was selected based on the highest F_1 score of 0.9857 (App. Table A.1). Protein-coding genes clustered into 29,461 homology groups where 6,580 (22.3%) are core, 11,057 (37.5%) accessory and 11,824 (40.2%) unique. An individual genome consists of 48.7-57.7% core genes, 1.2-15.6% unique genes with the rest being accessory genes. The alpha value of Heaps' law was 0.41, indicating the pangenome is open (App. Fig. A.1 & A.2). GO enrichment analysis on the set of unique genes in each genome revealed a wide variety of enriched functions: regulation of gene expression, recombination, tissue development and cell transport. Most striking was the highly over-represented (32 observed against 2 expected) GO function "hemolysis in other organism" (GO:0044179) in *D. ananassae*. Hemolysis is the premature destruction of red blood cells to release their contents. This process can be invoked by pathogenic bacteria and fungi; moreover, many fly species function as vector for these pathogens [34, 35]. For this reason, we suspect that either the functional annotations were incorrectly assigned or the sample was contaminated.

Four phylogenetic trees (core SNP, consensus, k-mer, gene) were inferred and compared to each other. The tree topology was identical for the core SNP phylogeny (Fig. 6A & App. Fig. A.3), the k-mer distance tree (App. Fig. A.4) and the consensus tree (App. Fig. A.5). These three trees are in agreement with literature, for instance the phylogeny reported by Paris *et al.* (2013) [36]. The gene distance tree (App. Fig. A.6) also clustered the genomes correctly; however, the branching becomes inconsistent with the other topologies after the delineation of *D. ananassae*.

3.2 *Arabidopsis thaliana*

To create a plant pangenome we selected *A. thaliana* (NCBI:txid3702) genomes from the study by Gan *et al.* in 2011 [3], together with the set by Jiao & Schneeberger (2020)[4] (Additional file 1, Table 2). The *Landsberg erecta* accession was present in both datasets, but only the most recent assembly was included to maintain a non-redundant pangenome. Furthermore, the *A. thaliana* reference genome Col-0 with the TAIR10 annotation was included, resulting in a total of 25 genomes. All genome assemblies are chromosome-level, with sizes ranging between 116.8 Mb and 120.8 Mb. The two datasets differ strongly in the number of annotated genes per genome; the seven accessions from Jiao & Schneeberger and Col-0 have around 27,000 genes, where the genomes from Gan *et al.* have around 30,000 genes. Only the longest gene transcripts were included for homology grouping and further analyses. All genomes have at least 98% complete and single-copy genes from the BUSCO *Brassicales* odb10 set.

We selected the d4 grouping (protein similarity of $\geq$ 65%) over d3 ($\geq$ 75%) based on the lowest number of false negatives (App. Table B.1). Proteins clustered into 41,801 homology groups: 22,758 (59.2%) groups were classified as core, 12,809 (33.3%) as accessory and 2894 (7.5%) as unique. *A. thaliana* genomes consists of 75.9-86.3% core genes with only 0.2-0.9% unique genes. The alpha value

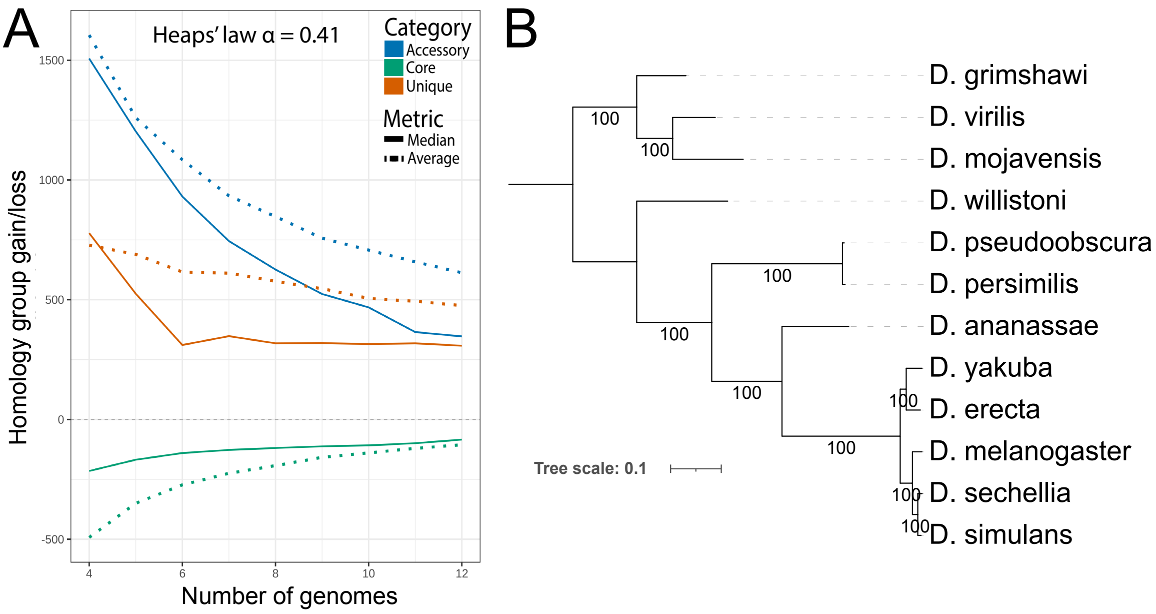

Figure 6: The change of homology group categories during pangenome structure estimation (A) and the core SNP phylogeny of the *Drospophila* genus pangenome (B). In agreement with the phylogeny of Paris *et al.* [36], the root is placed between *D. willstoni* and the clade of *D.grimshawi*, *D.virilis* and *D. mojavensis*. Values on branches represent bootstrap support values obtained through 10,000 bootstrap replications.

of Heaps' law was 1.22, indicating the pangenome is closed (App. Fig. B.1 & B.2). A visualization of the pangenome growth simulation (Fig. 7A) shows unique genes are lost when increasing the number of genomes. Pfam domains were classified as 92.1% core, 6.7% accessory and 1.2% unique. The majority (43) of unique Pfam domains were found in Col-0, with only 12 unique domains distributed over the other accessions. This uneven distribution is most likely an effect of the high quality annotation of Col-0.

All four phylogenies (core SNP, consensus, k-mer, gene) show a clear separation of accessions that corresponds with the two datasets. The high-resolution core SNP tree was inferred from 267,146 parsimony informative SNPs derived from 21,841 single-copy groups (Fig. 7B, App. Fig. B.3). As the SNP tree was inferred from a high number of sites, the low bootstrap values on several internal nodes are more likely to indicate inconsistency in the phylogenetic signal, possibly due to recombination, rather than insufficient data. The split network of core SNP tree visualizes the unresolved relationships in three clades (App. Fig. B.4). The clustering in the core SNP tree, k-mer tree (App. Fig. B.5) and the consensus tree (App. Fig. B.6) was almost identical, except for the placement of Can-0. In the core SNP tree Can-0 (from Gan) was placed in a clade with Edi-0, Bur-0, Zu-0 and Sf-2, where in the consensus tree it serves as outgroup of the clade of Jiao & Schneeberger and the k-mer tree places it in the latter clade. The gene distance phylogeny (App. Fig. B.7) stood out as Col-0 clustered into the Jiao & Schneeberger clade, where in the other trees it was placed in the Gan clade.

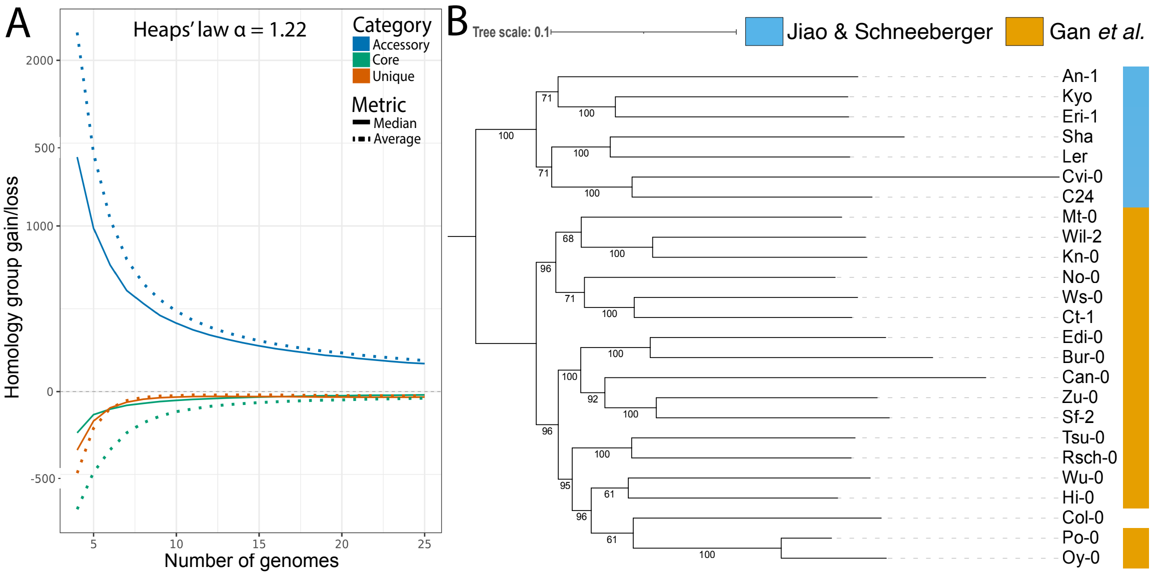

Figure 7: The change of homology group categories during pangenome structure estimation (A) and the core SNP phylogeny (B) of the *A. thaliana* pangenome. The root is placed at the midpoint of the tree. Bootstrap values are displayed on the branches.

3.3 *Saccharomyces cerevisiae*

We built a model fungal species-level pangenome of 100 *S. cerevisiae* (NCBI:txid4932) strains in five populations with different geographic and environmental origin data, obtained from the study of Strope *et al.*, in 2015 [5] (Additional file 1, Table 3). No genome annotations were available for strains SK1, Σ1278b, YPS163, and M22; nevertheless, their genome assemblies were included in the pangenome construction. Genome assembly sizes varied from 11.7 to 13.5 Mbp, the number of protein-coding genes ranged between 5230 and 6009, and the percentage of GC content differs from 38.1% to 38.9%. The class level BUSCO set *Saccharomycetes* odb10 was used to determine the optimal homology grouping.

The grouping of proteins was highly robust, showing no changes in the 0.9996 F_1 score for parameter settings d3-d7 (App. Table C.1). Based on the stringency and the highest score, the d3 grouping with 6,235 homology groups was selected for the analysis. The majority of groups (53.1%) were classified as accessory, followed by 39.1% core groups and 7.8% unique groups. Six strains did not have any unique genes, causing a noticeable difference in the proportion of unique gene content, ranging from 0% to 2.8%. The alpha value of Heaps' law was 0.89, indicating the pangenome can be considered open (App. Fig. C.1 & C.2). This is illustrated by the visualization of the number of classified groups (Fig. 8A). The gene content of the *S. cerevisiae* reference genome (S288c) clustered in 5,759 groups, leaving 476 groups groups with genes that have less than 75% sequence similarity to the reference genes. This is in line with the 401 non-reference genes reported in [5]. Several phenotype exclusive groups were identified when using strain ancestry as a phenotype: 162 Wine/Europe, 9 North American, 4 West African, and 4 Sake groups. The phenotype exclusive group from all ancestries, except the North American, were enriched for GO terms related to biosynthetic processes. This indicates that strains from different population ancestries potentially encode for their own biosynthetic pathways in order to produce secondary (specialized) metabolites [37].

We applied the four phylogenetic methods (core SNP, consensus, k-mer, gene) to the *S. cerevisiae* pangenome. Only the gene distance method (App. Fig. C.7) failed to cluster strains from the same ancestry and placed them into separate clades. We compared PanTools' k-mer tree (Fig. 8B, App. Fig. C.5) to Strope's SNP phylogeny (of a 218-Kb region) and observed the clustering of populations was nearly identical. The consensus phylogeny (App. Fig. C.6) was distinctive as it was the only tree where all European strains clustered together without additional mosaic strains. The core-SNP tree (App. Fig. C.3, & C.4) was inferred from 73,671 SNPs that were well supported except for three splits. S288c is placed within the European clade, while it serves as outgroup of this clade in the k-mer tree, the consensus tree, and Strope's SNP tree.

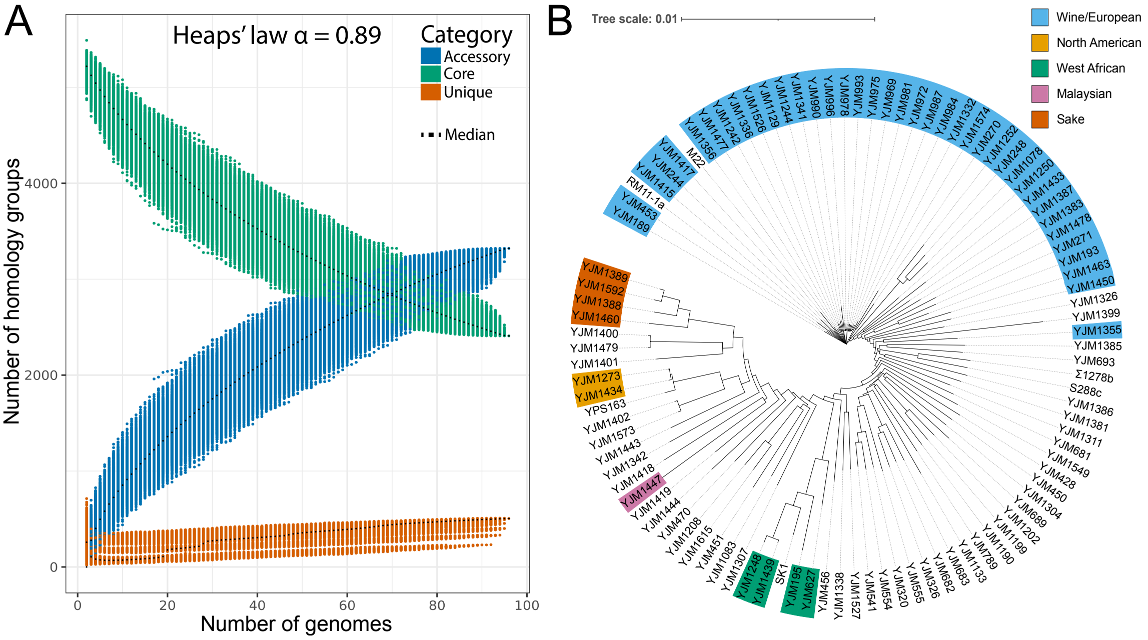

Figure 8: *S. cerevisiae* pangenome: structure simulation of 10,000 random genome combinations (A) and an unrooted k-mer distance tree (B). Branch colours correspond to one of the five strain populations described by Strope *et al.* [5].

3.4 *Pectobacterium*

In earlier work, we performed a comprehensive pangenomic analysis of the *Pectobacterium* genus (NCBI:txid122277), a diverse group of gram-negative bacteria causing soft rot disease in numerous plant hosts [1]. Supporting figures of the analysis are included in the previous manuscript [1]: Heaps's law visualization is part of Figure 1 of the main article and the phylogenetic trees are figures S8-S12 of Additional file 2. The pangenome was constructed from a set of novel genomes combined with publicly available data, resulting in 197 genomes of 19 different species (Additional file 1, Table 4). All genomes were (re)annotated and have between 3944 to 4719 protein-coding genes. Protein sequences clustered into 22,347 homology groups with the d4 ($\geq$ 65%) setting; 2032 (9.1%) groups were categorized as core, 13,168 (58.9%) as accessory and 7147 (32%) as unique. The average *Pectobacterium* genome consists of 47% core genes, 1% unique genes and 52% accessory genes. The alpha value of Heaps' law was 0.53, indicating an open pangenome structure. This is as

expected for a genus-level pangenome of prokaryotes, that generally have an extensive exchange of genetic information within populations. Field bioassays were performed over two consecutive years to estimate strain virulence. This information was incorporated into the pangenome by annotating genomes as: 'virulent', 'avirulent' or 'unknown'. A total of 86 homology groups were associated with the virulent phenotype. Furthermore, species names were used as a phenotype to find species-specific and exclusive genes. However, we could only identify genes for species that were represented by a low number of genomes as well as being highly similar to each other. This leads us to believe that the concept of species-specific genes, genes specific to a clade or lineage, does not apply to the *Pectobacterium* genus.

In the publication, we constructed phylogenetic trees using five distinct methods (core SNP, k-mer, gene, MLSA, ANI) and compared their topologies with the IQ-tree AU-test. The core SNP tree and k-mer distance tree were not significantly different. For this use case, we inferred a consensus tree based on 24,645 ML gene trees (Fig. 9). The tree shows the correct clustering and the branching order is highly similar to the core SNP and k-mer tree. However, NAK 467 clustered separately from the *P. aquaticum* clade, when in the other five trees it was placed inside the clade.

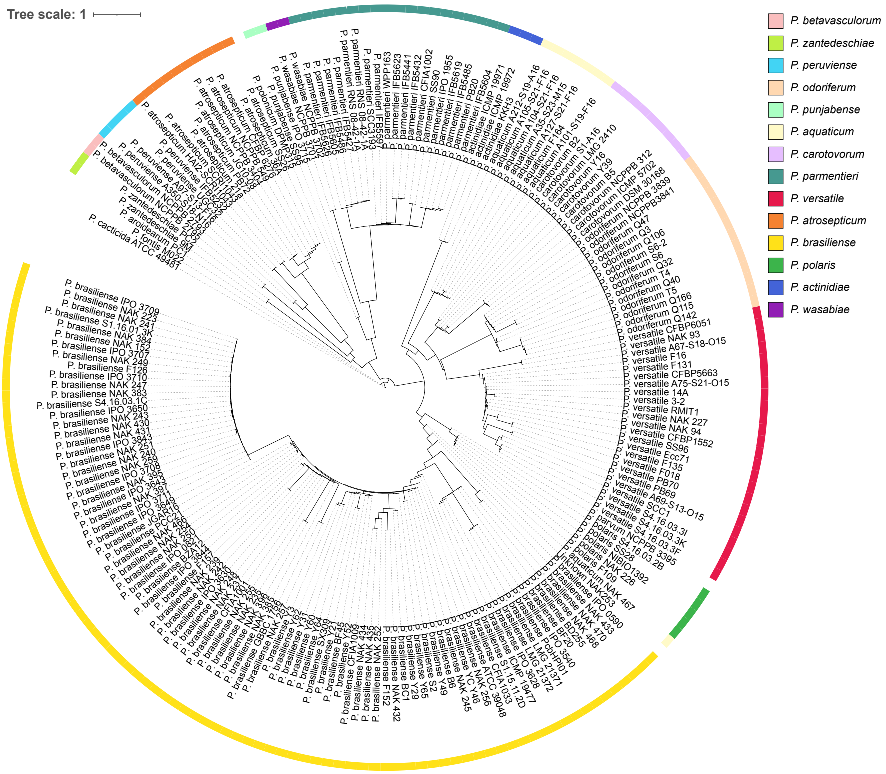

Figure 9: Consensus tree of 197 *Pectobacterium* genomes. The tree was rooted using *P. cacticida* ATCC 49481 as outgroup.

3.5 SARS-CoV-2

For the virus use case we created a pangenome of SARS-CoV-2, the virus responsible for the worldwide Covid-19 pandemic. First, we downloaded all available SARS-CoV-2 (NCBI:txid2697049) genomes from NCBI Datasets (https://www.ncbi.nlm.nih.gov/datasets/) on October 2021. Pangolin (v3.14) [38] was used to assign lineages according to the Pango nomenclature [39]; PangoLEARN was updated to the most recent version on October 11 2021. We randomly selected 10,000 genomes from parental lineages and variants that are currently being monitored around the world: A, A.1, AY.4 (Delta), B.1, B.1.1.7 (Alpha), B.1.351 (Beta), B.1.427 (Epsilon), B.1.429 (Epsilon), B.1.525 (Eta), B.1.526 (Iota), B.1.617.1 (Kappa), B.1.617.2 (Delta), P.1 (Gamma), and P.2 (Zeta) (Additional file 1, Table 5). For a genome to be selected, up to 1% of nucleotides were allowed to be unknown or ambiguous. The number of variants in the selection of 10,000 genomes is unevenly balanced due to the limited availability of high quality strains in the NCBI dataset. The SARS-CoV-2 reference genome (NC_045512.2) was included as final addition to the pangenome. All genomes consist of a single sequence, the length ranging between 29.41 Kb to 30.02 Kb and GC in the range 37.88-38.11%.

Because the reference genome was the only annotated genome, we limited the analysis to pangenome construction and estimation of the k-mer distances between genomes. Constructing a reliable phylogeny for SARS-CoV-2 genomes is known to be difficult due to a low number of distinct site patterns with a large number of lineages [40]. PanTools' k-mer method was able to distinguish the variants and clustered them into separate clades (Fig. 10). Moreover, the tree also shows a correct clustering of parental lineages A, A.1, and B, with a few misclassifications. The B.1 lineage is scattered throughout the tree but this can be explained by it being the parental lineage of the selected variants. The placement of AY.4 strains within B.1.617.2 is correct, as it is a subvariant of the Delta variant.

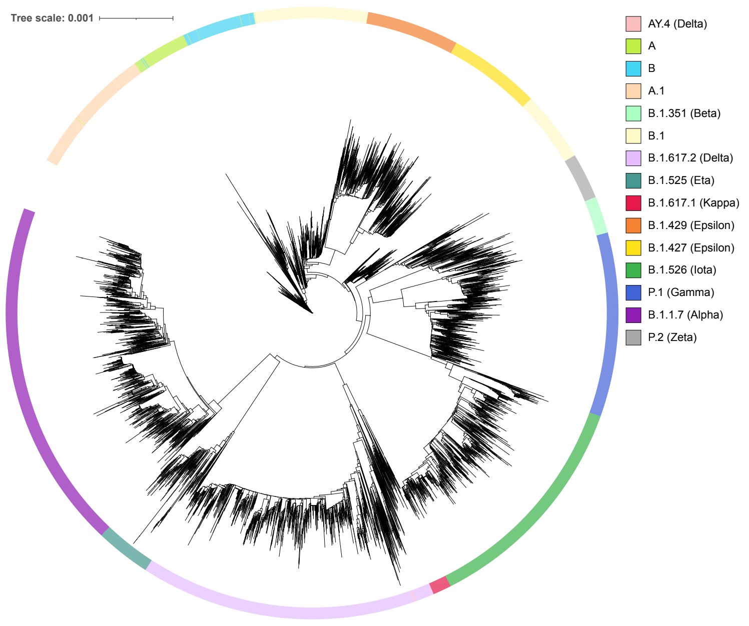

Figure 10: Unrooted k-mer distance tree of 10,000 SARS-CoV-2 genomes. The colors in the ring surrounding the tree represent the Pangolin lineages.

We visually compared our k-mer tree to the real-time and regularly updated Nextstrain phylogeny (see App. Fig. D.1) (nextstrain.org). The tree structures agree for the most part, with a few inconsistencies. For instance, the k-mer tree did not have the Epsilon clade between Iota and Beta. In conclusion, the k-mer method is able to classify variants with high accuracy, but the tree does not reflect the actual phylogeny due to the incorrect branching order.

4 Scalability

In this section we test PanTools' current scalabilty to *H. sapiens* (human) and *S. lycopersicum* (tomato) and discuss bottlenecks in the construction algorithm and upcoming solutions.

4.1 Scalability use cases

The largest input of genome sequences from the use cases was *A. thaliana* with a combined size of 2.96 Gb. These genomes were quite repetitive as we observed transposable elements account for 20% of the genomes in the TAIR10 annotation of the Col-0 accession. To demonstrate the current construction algorithm's scalability we applied PanTools to larger and repetitive tomato and human genomes. The graph building was carried out on multiple distinct Linux servers to assess variability in runtime: Intel(R) Xeon(R) CPU E5-2690 v4 @ 2.60GHz (56 CPUs, 252 GB RAM), Intel(R) Xeon(R) CPU E5-2670 v3 @ 2.30GHz (48 CPUs, 378 GB RAM) and AMD EPYC 7532 32-Core Processor (128 CPUs, 2.9 TB RAM). A maximum heap size of 120 GB was assigned to the JVM. Because the graph construction is highly dependent on I/O operations, experiments were performed on local disks for the lowest possible disk latency. The choice of k is crucial for the construction and analysis; a very low value of k tangles the graph, but increasing k too much leads to disconnected subgraphs. Two pangenomes were constructed for selected k-mer sizes, a third run was performed when run times were highly divergent.

We built the tomato pangenome from 13 chromosome-scale tomato genomes made available by the groups of M. Schatz and Z. Lippman (https://solgenomics.net/projects/tomato13) (Additional file 1, Table 6). The assembly sizes varied between 774 Mb and 792 Mb, the combined input size for the pangenome was 10.2 Gb. Repeat content was expected to be in line with the 63.28% of the tomato reference 'Heinz 1706' [41]. The average construction time (wall-clock) of the tomato pangenome using an (optimal) k of 19 was around 96 hours (Fig. 11). Peak memory usage for the construction was ~63 GB. Increasing the k-mer size to 21, lowered the runtime almost by 25 hours. Further increasing k to 31 halved the original runtime to approximately 50 hours.

The human pangenome was constructed from five genomes (GRCh38.p14, HuRef, CHM1_1.1, T2T-CHM13v2.0, YH_2.0) ranging between 2.5 to 3.1 Gb in genome size, to a combined size of 14.5 Gb (Additional file 1, Table 7). Around 50% of the first version of the human reference genome (GRCh37) is made up of repetitive sequences [42]. The average construction runtime was 125 hours for the calculated optimal k-mer size of 21 (Fig. 11). The maximum reported memory was ~94Gb. We observed the localization step for the last genome almost took half of total runtime. The construction using longer k-mers of 31 and 41 resulted in an average runtime of 75 and 56, respectively.

4.2 Bottleneck in the construction algorithm

Genome size, fragmentation, variability and repetitiveness are key factors behind the computational challenge of pangenome construction. PanTools v3 uses the exact same graph

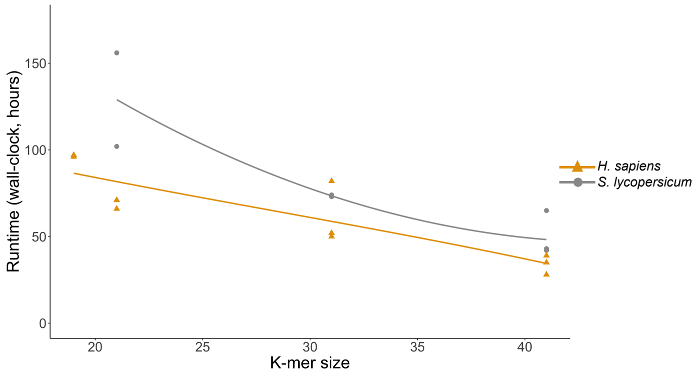

Figure 11: Influence of k-mer size on the graph construction time of the *H. sapiens* (grey) and *S. lycopersicum* (orange) pangenome.

construction algorithm we introduced in 2016. With the increase in high-quality genomes, we observed the current limitations of the tool and identified multiple bottlenecks in the algorithm.

PanTools' graph construction algorithm consists of two parts: graph construction and localization. In the initial step, KMC is employed to create the k-mer sequences together with a k-mer index used to construct a compressed de Bruijn graph. After the graph is built, localization starts and genomic coordinates are added to the graph. During localization we go over every sequence to determine the k-mer positions and the edge properties, where the coordinates are stored, are directly updated. The current method for storage, in large arrays, causes bottlenecks in case of highly frequent k-mers. In large repetitive genomes we observed the majority of time was spent on storing the genomic coordinates. For the constructed human pangenome ($k = 21$) 1126 k-mer nodes had a frequency over 100,000 of which 18 nodes had a frequency over 1 million. The k-mer consisting of only A's or (T's) was visited the most, having a frequency of nearly 3.5 million (Fig. 12).

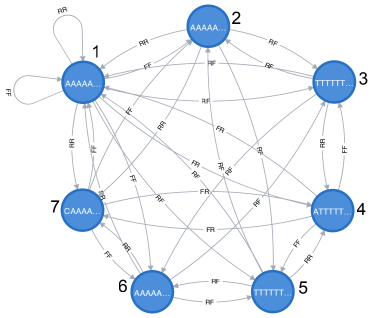

	K-mer	Reverse complement	Frequency
1	AAAAAAAAAAAAAAAAAAAAA	TTTTTTTTTTTTTTTTTTTTT	3,461,243
2	AAAAAAAAAAAAAAAAAAAAC	GTTTTTTTTTTTTTTTTTTTT	104,067
3	TTTTTTTTTTTTTTTTTTTTA	TAAAAAAAAAAAAAAAAAAAA	130,482
4	ATTTTTTTTTTTTTTTTTTTT	AAAAAAAAAAAAAAAAAAAAT	184,772
5	TTTTTTTTTTTTTTTTTTTTC	GAAAAAAAAAAAAAAAAAAAA	44,282
6	AAAAAAAAAAAAAAAAAAAAG	CTTTTTTTTTTTTTTTTTTTT	528,650
7	CAAAAAAAAAAAAAAAAAAAA	TTTTTTTTTTTTTTTTTTTTG	643,548

Figure 12: The most frequently occurring k-mer in the human pangenome. Over 99.9% of the edges are directed to itself or one letter variations.

We are working on a redesign of the construction algorithm to overcome this issue. Here, we already discuss two changes to be introduced in the next major PanTools version. First, the implementation of a cache, a storage layer keeping coordinates of high-frequent k-mers in memory, only writing coordinates to the graph when clearing the cache. Second, parallelizing the

localization enables multiple sequences to be indexed simultaneously. This is still a single process thread, localizing a single sequence a time.

5 Data availability

PanTools v3 is available at https://git.wur.nl/bioinformatics/pantools, released under the GNU GPLv3 license. We developed a Snakemake pipeline [43] that reproduces the pangenome analyses on the seven presented datasets. For the five use cases, the datasets including the genomes, structural annotations, InterProscan annotations and eggnog annotations are enclosed in the download. The genome assemblies for *S. lycopersicum*, and *H. sapiens* required for the scalabilty use case are downloaded separately following included instructions. The Snakemake pipeline, together with instructions and data to run it, is available at https://doi.org/10.4121/19874485.

Additional file 1: Table 1-7. Genome and annotation statistics of *Drososophila*, *A. thaliana*, *S. cerevisiae*, *Pectobacterium*, SARS-CoV-2, *S. lycopersicum*, and *H. sapiens* datasets.

6 Installation

PanTools requires (a minimum of) Java 8, KMC 2.3 [44] and MCL 14 [45] for pangenome construction, annotation, and homology grouping. Downstream analysis functionalities may require a variety of dependencies that can be installed manually or through Conda [46]. In our installation manual we provide instructions to create a Conda environment for PanTools with all dependencies. To be able to open, browse and query a pangenome database, the Neo4j 3.5.30 community edition (https://neo4j.com/download-center) is required, which must be installed manually. The required functional databases Gene Ontology (GO), Pfam, TIGRFAM are already included in the Git repository. The InterPro database is downloaded upon the first time functional annotations are added to the pangenome. More information regarding the installation can be found in the online documentation at https://git.wur.nl/bioinformatics/pantools.

References

[1] Jonkheer EM, Brankovics B, Houwers IM, *et al.* The Pectobacterium pangenome, with a focus on Pectobacterium brasiliense, shows a robust core and extensive exchange of genes from a shared gene pool. *BMC Genomics*, 22:265, 12 2021.

[2] Larkin A, Marygold SJ, Antonazzo G, *et al.* FlyBase: updates to the Drosophila melanogaster knowledge base. *Nucleic Acids Research*, 49:D899–D907, 1 2021.

[3] Gan X, Stegle O, Behr J, *et al.* Multiple reference genomes and transcriptomes for Arabidopsis thaliana. *Nature*, 477:419–423, 9 2011.

[4] Jiao WB, Schneeberger K. Chromosome-level assemblies of multiple Arabidopsis genomes reveal hotspots of rearrangements with altered evolutionary dynamics. *Nature Communications*, 2020.

[5] Strope PK, Skelly DA, Kozmin SG, *et al.* The 100-genomes strains, an S. cerevisiae resource that illuminates its natural phenotypic and genotypic variation and emergence as an opportunistic pathogen. *Genome Research*, 25:762–774, 5 2015.

[6] Sheikhizadeh S, Schranz ME, Akdel M, *et al.* PanTools: representation, storage and exploration of pan-genomic data. *Bioinformatics*, 32:i487–i493, 9 2016.

[7] Sheikhizadeh Anari S, de Ridder D, Schranz ME, *et al.* Efficient inference of homologs in large eukaryotic pan-proteomes. *BMC Bioinformatics*, 19:340, 12 2018.

[8] Carbon S, Douglass E, Good BM, *et al.* The Gene Ontology resource: enriching a GOld mine. *Nucleic Acids Research*, 49:D325–D334, 1 2021.

[9] Mistry J, Chuguransky S, Williams L, *et al.* Pfam: The protein families database in 2021. *Nucleic Acids Research*, 2021.

[10] Blum M, Chang HY, Chuguransky S, *et al.* The InterPro protein families and domains database: 20 years on. *Nucleic Acids Research*, 49:D344–D354, 1 2021.

[11] Li W, O'Neill KR, Haft DH, *et al.* Refseq: Expanding the prokaryotic genome annotation pipeline reach with protein family model curation. *Nucleic Acids Research*, 2021.

[12] Galperin MY, Wolf YI, Makarova KS, *et al.* COG database update: Focus on microbial diversity, model organisms, and widespread pathogens. *Nucleic Acids Research*, 2021.

[13] Käll L, Krogh A, Sonnhammer ELL. An HMM posterior decoder for sequence feature prediction that includes homology information. *Bioinformatics*, 21:i251–i257, 6 2005.

[14] Armenteros JJA, Tsirigos KD, Sønderby CK, *et al.* Signalp 5.0 improves signal peptide predictions using deep neural networks. *Nature Biotechnology*, 37:420–423, 4 2019.

[15] Jones P, Binns D, Chang HY, *et al.* InterProScan 5: genome-scale protein function classification. *Bioinformatics*, 30:1236–1240, 5 2014.

[16] Cantalapiedra CP, Hernández-Plaza A, Letunic I, *et al.* eggNOG-mapper v2: Functional Annotation, Orthology Assignments, and Domain Prediction at the Metagenomic Scale. *Molecular Biology and Evolution*, 2021.

[17] Bland JM, Altman DG. Multiple significance tests: the Bonferroni method. *BMJ*, 310:170, 1995.

[18] Benjamini Y, Hochberg Y. Controlling the False Discovery Rate: a Practical and Powerful Approach to Multiple Testing. *Journal of the Royal Statistical Society. Series B (Methological)*, 57(1):289–300, 1995.

[19] Ellson J, Gansner E, Koutsofios L, *et al.* Graphviz: Open source graph drawing tools. In Mutzel P, Jünger M, Leipert S, editors, *Graph Drawing. GD 2001. Lecture Notes in Computer Science.*, volume 2265 LNCS, pages 483–484. 2002.

[20] Waterhouse RM, Seppey M, Simão FA, *et al.* BUSCO applications from quality assessments to gene prediction and phylogenomics. *Molecular Biology and Evolution*, 35:543–548, 3 2018.

[21] Guimarães LC, de Jesus LB, Viana MVC, *et al.* Inside the pan-genome - methods and software overview. *Current Genomics*, 16:245–252, 2015.

[22] Tettelin H, Riley D, Cattuto C, *et al.* Comparative genomics: the bacterial pan-genome. *Current Opinion in Microbiology*, 11:472–477, 10 2008.

[23] Nakamura T, Yamada KD, Tomii K, *et al.* Parallelization of MAFFT for large-scale multiple sequence alignments. *Bioinformatics*, 34:2490–2492, 7 2018.

[24] Price MN, Dehal PS, Arkin AP. FastTree 2 approximately Maximum-Likelihood trees for large alignments. *PLoS ONE*, 5:e9490, 3 2010.

[25] Nguyen LT, Schmidt HA, von Haeseler A, *et al.* IQ-TREE: A fast and effective stochastic algorithm for estimating Maximum-Likelihood phylogenies. *Molecular Biology and Evolution*, 32:268–274, 1 2015.

[26] Glaeser SP, Kämpfer P. Multilocus sequence analysis (MLSA) in prokaryotic taxonomy. *Systematic and Applied Microbiology*, 38:237–245, 6 2015.

[27] Sung WK. Greedy Consensus Tree and Maximum Greedy Consensus Tree Problems. In *Lecture Notes in Computer Science (including subseries Lecture Notes in Artificial Intelligence and Lecture Notes in Bioinformatics)*. 2019. ISBN 9783030105631.

[28] Zhang C, Scornavacca C, Molloy EK, *et al.* ASTRAL-pro: Quartet-based species-tree inference despite paralogy. *Molecular Biology and Evolution*, 37:3292–3307, 11 2020.

[29] Ondov BD, Treangen TJ, Melsted P, *et al.* Mash: Fast genome and metagenome distance estimation using Minhash. *Genome Biology*, 2016.

[30] Paradis E, Schliep K. ape 5.0: an environment for modern phylogenetics and evolutionary analyses in r. *Bioinformatics*, 35:526–528, 2 2019.

[31] Goris J, Konstantinidis KT, Klappenbach JA, *et al.* DNA-DNA hybridization values and their relationship to whole-genome sequence similarities. *International Journal of Systematic and Evolutionary Microbiology*, 57:81–91, 1 2007.

[32] Jain C, Rodriguez-R LM, Phillippy AM, *et al.* High throughput ANI analysis of 90k prokaryotic genomes reveals clear species boundaries. *Nature Communications*, 9:5114, 12 2018.

[33] Letunic I, Bork P. Interactive tree of life (iTOL) v5: an online tool for phylogenetic tree display and annotation. *Nucleic Acids Research*, 49:W293–W296, 7 2021.

[34] Junqueira ACM, Ratan A, Acerbi E, *et al.* The microbiomes of blowflies and houseflies as bacterial transmission reservoirs. *Scientific Reports*, 2017.

[35] Ramírez-Camejo LA, Maldonado-Morales G, Bayman P. Differential Microbial Diversity in Drosophila melanogaster: Are Fruit Flies Potential Vectors of Opportunistic Pathogens? *International Journal of Microbiology*, 2017.

[36] Paris M, Kaplan T, Li XY, *et al.* Extensive divergence of transcription factor binding in Drosophila embryos with highly conserved gene expression. *PLoS Genetics*, 2013.

[37] Katz L, Baltz RH. Natural product discovery: past, present, and future. *Journal of Industrial Microbiology and Biotechnology*, 43:155–176, 3 2016.

[38] O'Toole Á, Scher E, Underwood A, *et al.* Assignment of epidemiological lineages in an emerging pandemic using the pangolin tool. *Virus Evolution*, 2021.

[39] Rambaut A, Holmes EC, O'Toole Á, *et al.* A dynamic nomenclature proposal for SARS-CoV-2 lineages to assist genomic epidemiology. *Nature Microbiology*, 2020.

[40] Morel B, Barbera P, Czech L, *et al.* Phylogenetic analysis of SARS-CoV-2 data is difficult. *Molecular Biology and Evolution*, 2021.

[41] The Tomato Genome Consortium. The tomato genome sequence provides insights into fleshy fruit evolution. *Nature*, 485:635–641, 2012.

[42] International Human Genome Sequencing Consortium. Initial sequencing and analysis of the human genome. *Nature*, 409:860–921, 2001.

[43] Mölder F, Jablonski KP, Letcher B, *et al.* Sustainable data analysis with Snakemake. *F1000Research*, 10:33, 1 2021.

[44] Kokot M, Długosz M, Deorowicz S. KMC 3: counting and manipulating k-mer statistics. *Bioinformatics*, 33:2759–2761, 9 2017.

[45] Enright AJ. An efficient algorithm for large-scale detection of protein families. *Nucleic Acids Research*, 30:1575–1584, 4 2002.

[46] Anaconda Inc. Anaconda software distribution, 2020.

A Appendix: Drosophila

Table A.1: Statistics for the optimal homology grouping analysis of 12 *Drosophila* genomes. A total of 2,075 BUSCO genes were included in the benchmark, as these were present and single-copy in all genomes. A correct group is defined as perfectly placing one BUSCO gene in a single-copy group.

**Relaxation mode**	**Minimum sequence similarity**	**Homology groups**	**Core groups**	**Single-copy groups**	**Unique groups**	**Correct groups**
D1	95	114,746	345	270	95,434	130
D2	85	81,091	1,545	1,183	58,049	597
D3	75	60,383	3,074	2,330	37,794	1,097
D4	65	46,217	4,537	3,371	25,352	1,444
D5	55	36,624	5,687	4,095	17,390	1,641
D6	45	29,461	6,580	4,460	11,824	1,672
D7	35	23,757	7,115	4,493	7,840	1,643

**Relaxation mode**	**True positives**	**False positives**	**False negatives**	**Recall**	**Precision**	**F_1 score**
D1	10,664	81	14,236	0.4283	0.9925	0.5983
D2	16,799	171	8,101	0.6747	0.9899	0.8024
D3	20,805	244	4,095	0.8355	0.9884	0.9056
D4	23,108	296	1,792	0.9280	0.9874	0.9568
D5	24,263	365	637	0.9744	0.9852	0.9798
D6	24,674	490	226	0.9909	0.9805	0.9857
D7	24,799	926	101	0.9959	0.9640	0.9797

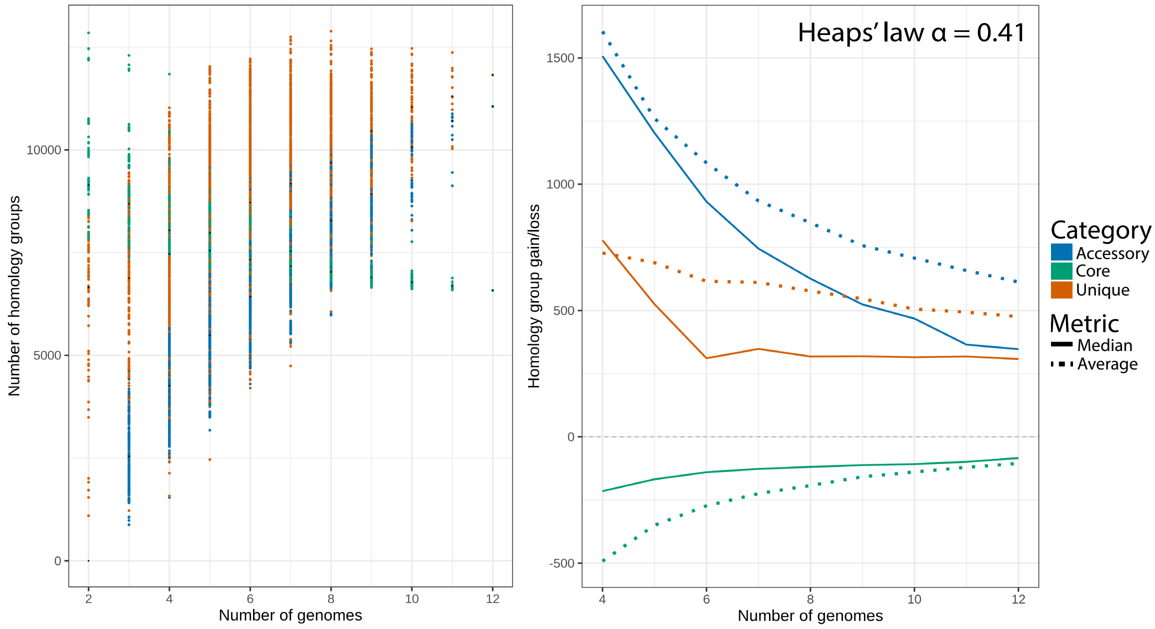

Figure A.1: Pangenome structure estimation of 12 *Drosophila* species using 10,000 random genome combinations (left plot) and the change of homology group categories during the simulation (right plot).

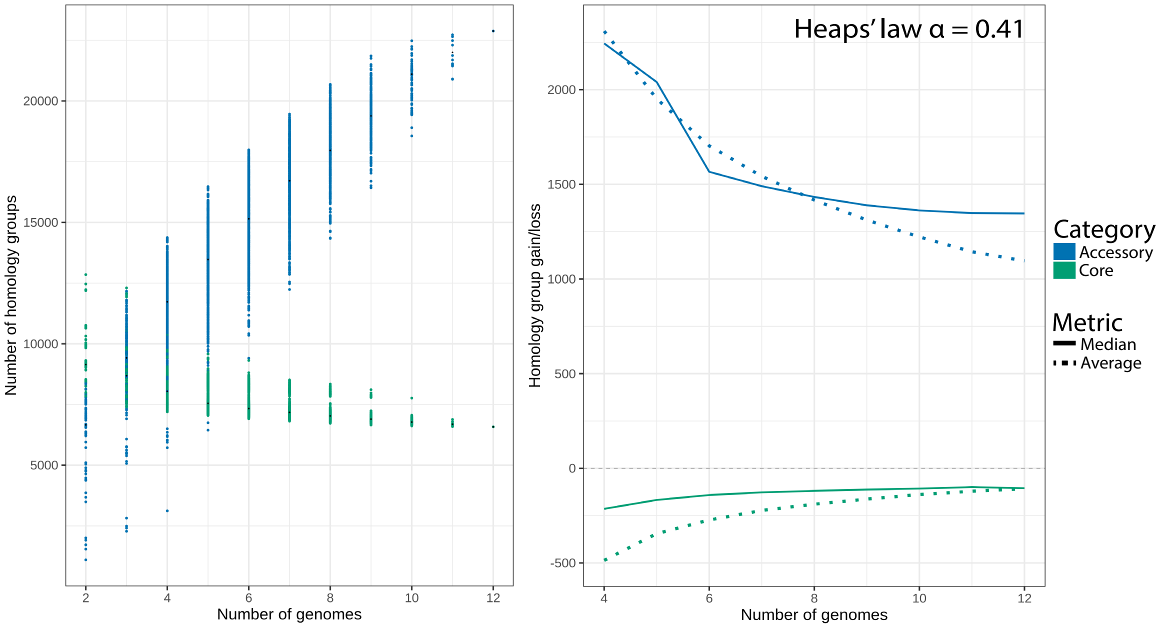

Figure A.2: Pangenome structure estimation of 12 *Drosophila* species using 10,000 random genome combinations (left plot) and the change of homology group categories during the simulation (right plot). The accessory category also includes unique homology groups.

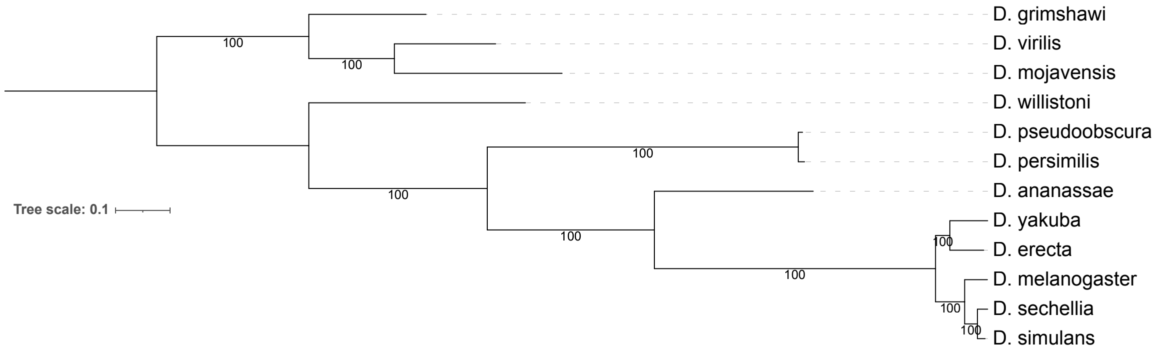

Figure A.3: Core SNP phylogeny of 12 *Drosophila* accessions. The phylogeny was inferred on 2,540,981 parsimony informative sites that were identified from 4,460 single-copy groups. Values on branches represent the bootstrap support obtained through 10,000 bootstrap replications. The root is placed between *D. willstoni* and the clade of *D.grimshawi*, *D.virilis* and *D. mojavensis.*

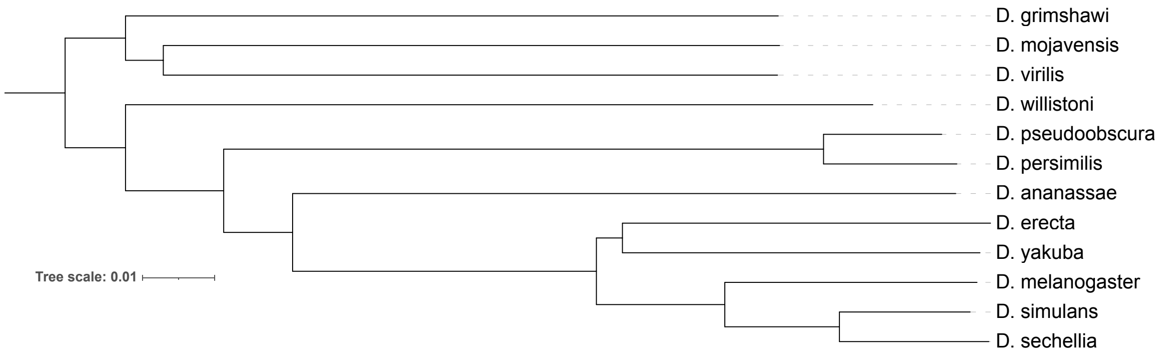

Figure A.4: *k*-mer distance tree of 12 *Drosophila* species. The phylogeny was inferred on pairwise distances calculated from 227.7 million *k*-mers. The root is placed between *D. willstoni* and the clade of *D.grimshawi*, *D.virilis* and *D. mojavensis.*

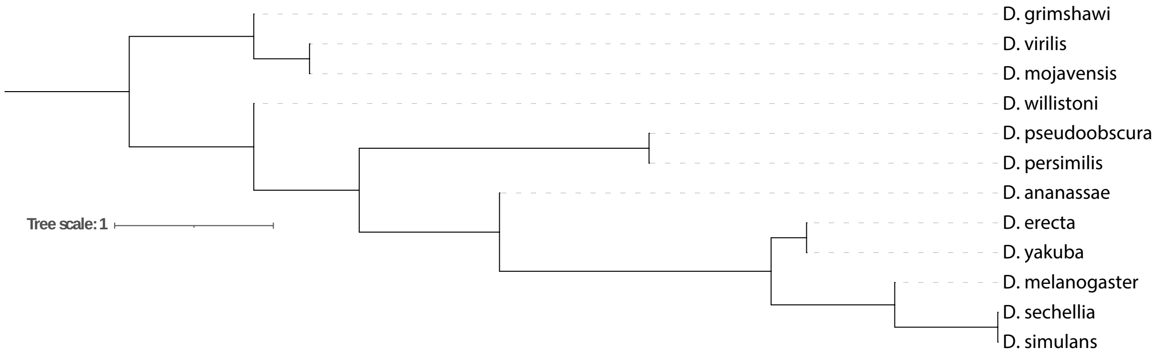

Figure A.5: Consensus tree of 12 *Drosophila* species. The phylogeny was inferred on 29,461 gene trees. The root is placed between *D. willstoni* and the clade of *D.grimshawi*, *D.virilis* and *D. mojavensis.*

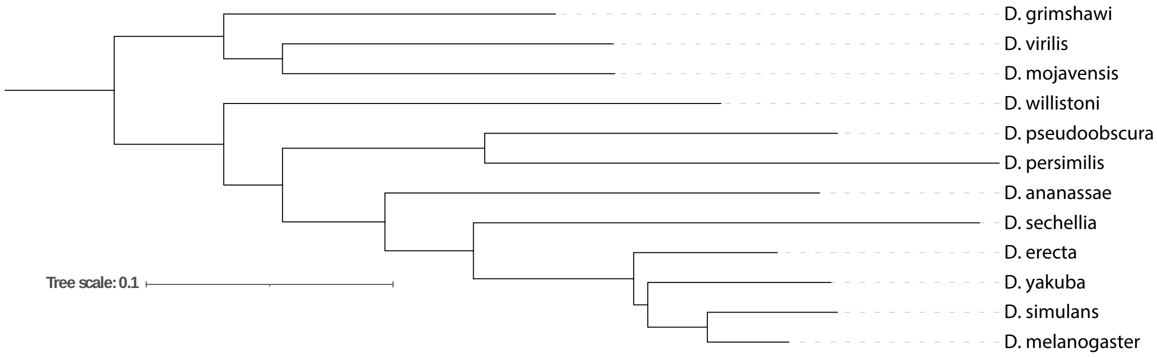

Figure A.6: Gene distance tree of 12 *Drosophila* accessions. The phylogeny was inferred on pairwise distances calculated from counting shared genes in 29,461 homology groups. The root is placed between *D. willstoni* and the clade of *D.grimshawi*, *D.virilis* and *D. mojavensis*.

B Appendix: *A. thaliana*

Table B.1: Statistics for the optimal homology grouping analysis of 25 *A. thaliana* genomes. A total of 4,299 BUSCO genes were included in the benchmark, as these were present and single-copy in all genomes. A correct group is defined as perfectly placing one BUSCO gene in a single-copy group.

Relaxation mode	Minimum sequence similarity	Homology groups	Core groups	Single-copy groups	Unique groups	Correct groups
D1	95	61,839	19,013	18,721	17,984	3,840
D2	85	46,142	21,811	21,302	7,153	4,208
D3	75	41,513	22,457	21,745	4,225	4,245
D4	65	38,461	22,758	21,841	2,894	4,244

Relaxation mode	True positives	False positives	False negatives	Recall	Precision	F_1 score
D1	105,085	26	2390	0.9778	0.9998	0.9886
D2	107,056	87	419	0.9961	0.9992	0.9976
D3	107,320	122	155	0.9986	0.9989	0.9987
D4	107,392	200	83	0.9992	0.9981	0.9987

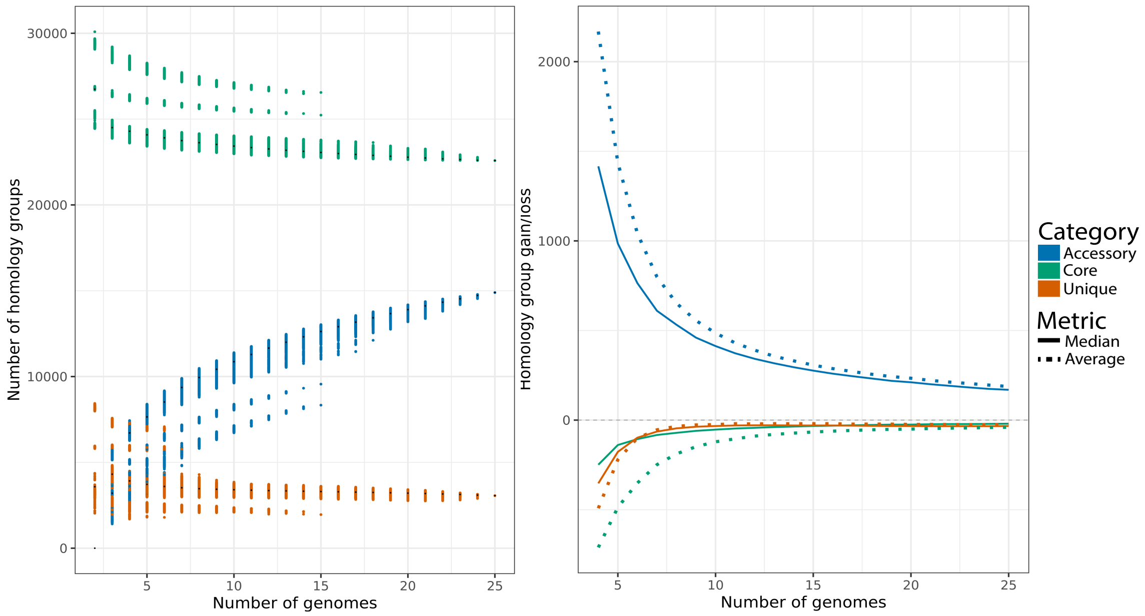

Figure B.1: Pangenome structure estimation of 25 *A. thaliana* accessions using 10,000 random genome combinations (left plot) and the change of homology group categories during the simulation (right plot).

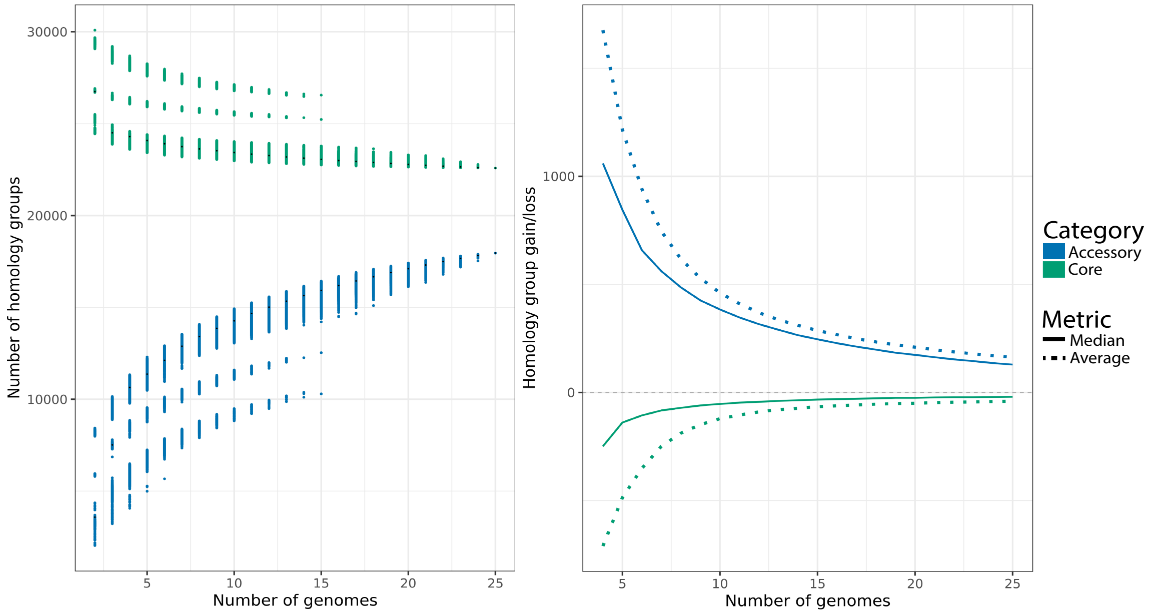

Figure B.2: Pangenome structure estimation of 25 *A. thaliana* accessions using 10,000 random genome combinations (left plot) and the change of homology group categories during the simulation (right plot). The accessory category also includes unique homology groups.

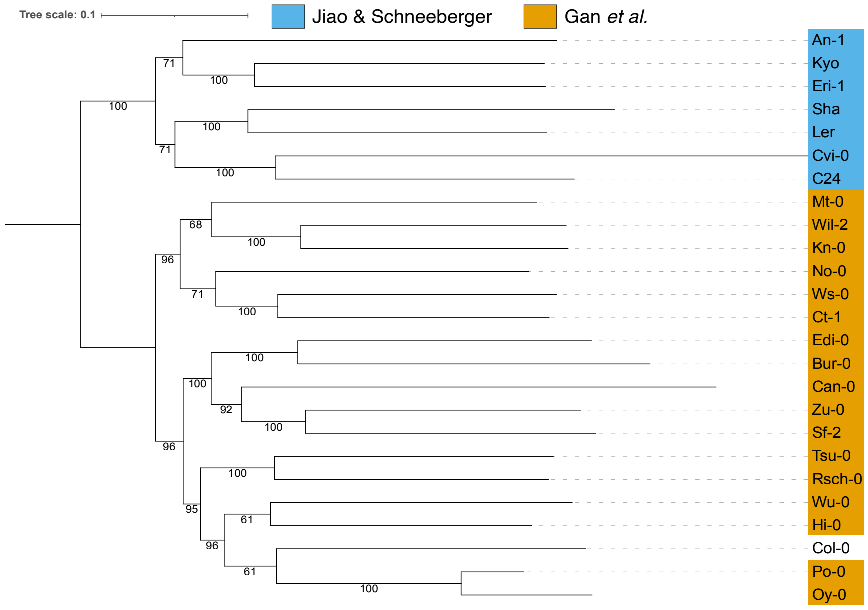

Figure B.3: Core SNP phylogeny of 25 *A. thaliana* accessions. The phylogeny was inferred on 267,147 parsimony informative sites that were identified from 21,841 single-copy groups. Values on branches represent the bootstrap support obtained through 10,000 bootstrap replications. The root is placed at the midpoint of the tree.

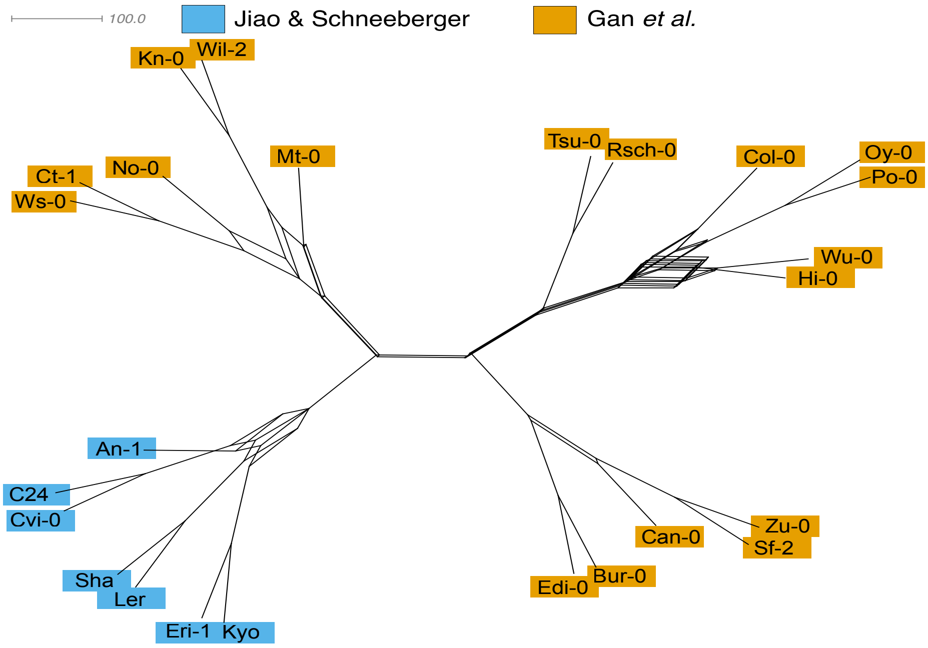

Figure B.4: Split network tree of 25 *A. thaliana* accessions. This is a different representation of the core SNP phylogeny, with every supported split being visualized.

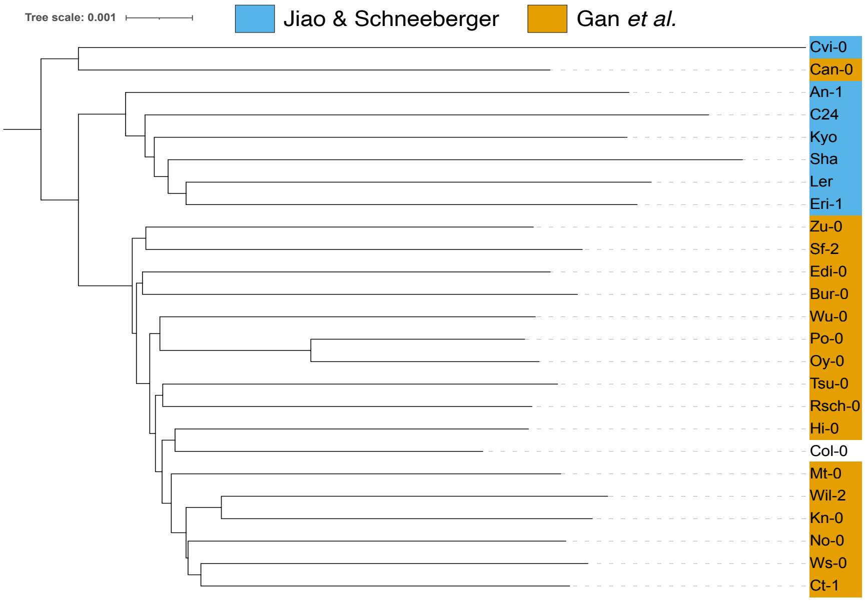

Figure B.5: k-mer distance tree of 25 *A. thaliana* accessions. The phylogeny was inferred on pairwise distances calculated from 149.2 million k-mers. The root is placed at the midpoint of the tree.

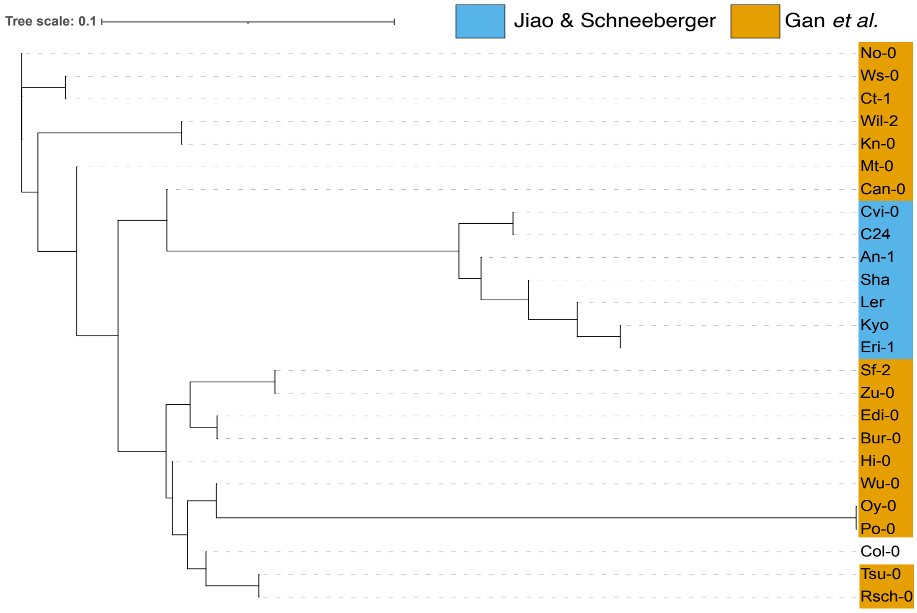

Figure B.6: Consensus tree of 25 *A. thaliana* accessions. The phylogeny was inferred on 38,461 gene trees. The root is placed at the midpoint of the tree.

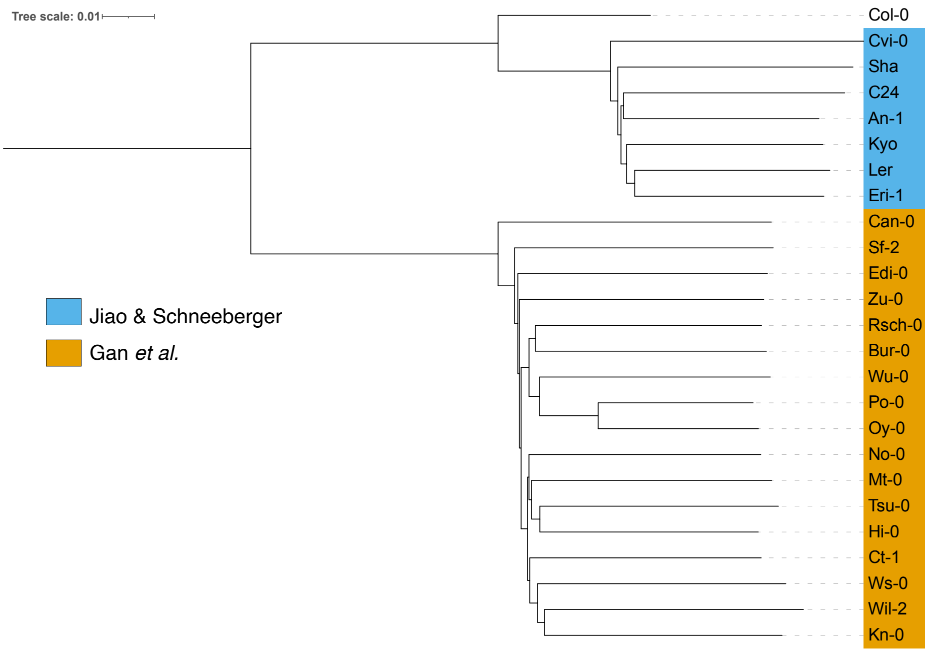

Figure B.7: Gene distance tree of 25 *A. thaliana* accessions. The phylogeny was inferred on pairwise distances calculated from counting shared genes in 38,461 homology groups. The root is placed at the midpoint of the tree.

C Appendix: *S. cerevisiae*

Table C.1: Statistics for the optimal homology grouping analysis of 96 *S. cerevisiae* genomes. A total of 1,062 BUSCO genes were included in the benchmark, as these were present and single-copy in all genomes. A correct group is defined as perfectly placing one BUSCO gene in a single-copy group.

**Relaxation mode**	**Minimum sequence similarity**	**Homology groups**	**Core groups**	**Single-copy groups**	**Unique groups**	**Correct groups**
D1	95	6,936	2,308	2,267	730	1,026
D2	85	6,372	2,395	2,333	558	1,048
D3	75	6,235	2,409	2,344	505	1,049
D4	65	6,167	2,412	2,340	479	1,049
D5	55	6,110	2,417	2,338	448	1,049
D6	45	5,994	2,421	2,311	411	1,049
D7	35	5,887	2,427	2,278	397	1,049
D8	35	5,483	2,412	2,014	382	1,022

**Relaxation mode**	**True positives**	**False positives**	**False negatives**	**Recall**	**Precision**	F_1 **score**
D1	101,847	11	105	0.9990	0.9999	0.9994
D2	101,893	11	62	0.9994	0.9999	0.9996
D3	101,893	11	59	0.9994	0.9999	0.9997
D4	101,893	11	59	0.9994	0.9999	0.9997
D5	101,893	11	59	0.9994	0.9999	0.9997
D6	101,893	11	59	0.9994	0.9999	0.9997
D7	101,893	11	59	0.9994	0.9999	0.9997
D8	101,895	5,785	57	0.9994	0.9463	0.9721

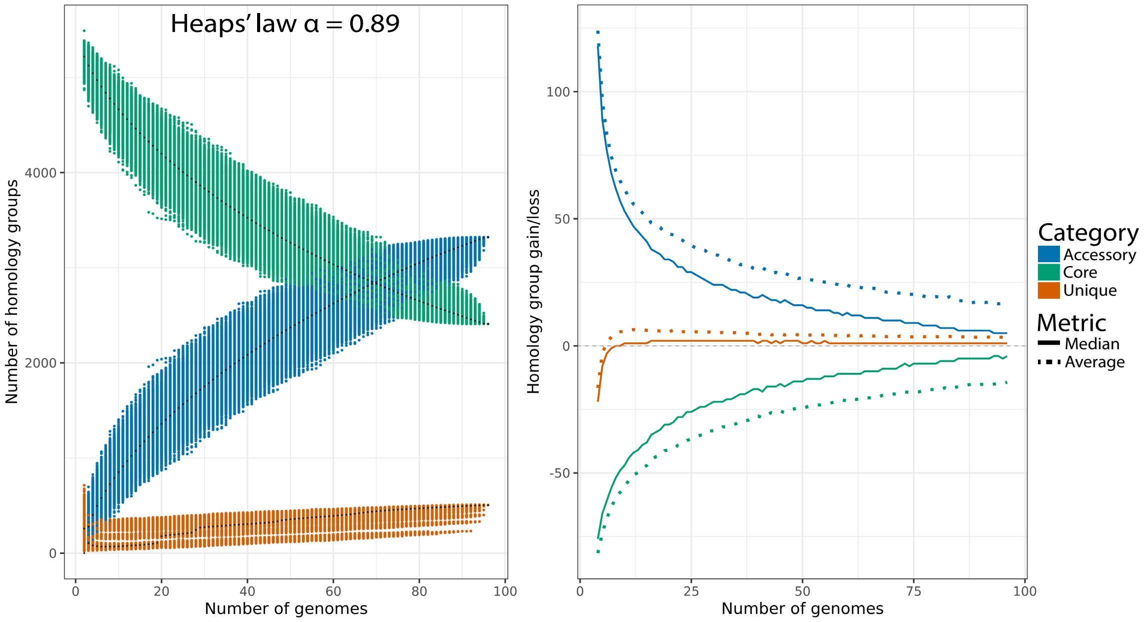

Figure C.1: Pangenome structure estimation of 96 *S. cerevisiae* strains using 10,000 random genome combinations (left plot) and the change of homology group categories during the simulation (right plot).

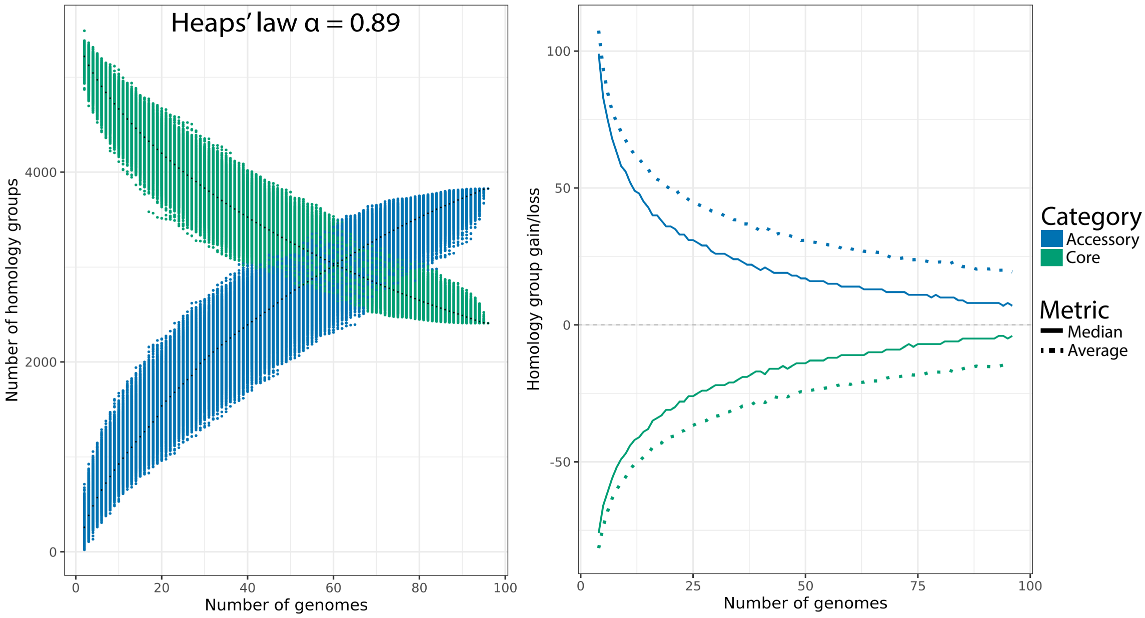

Figure C.2: Pangenome structure estimation of 96 *S. cerevisiae* strains using 10,000 random genome combinations (left plot) and the change of homology group categories during the simulation (right plot). The accessory category also includes unique homology groups.

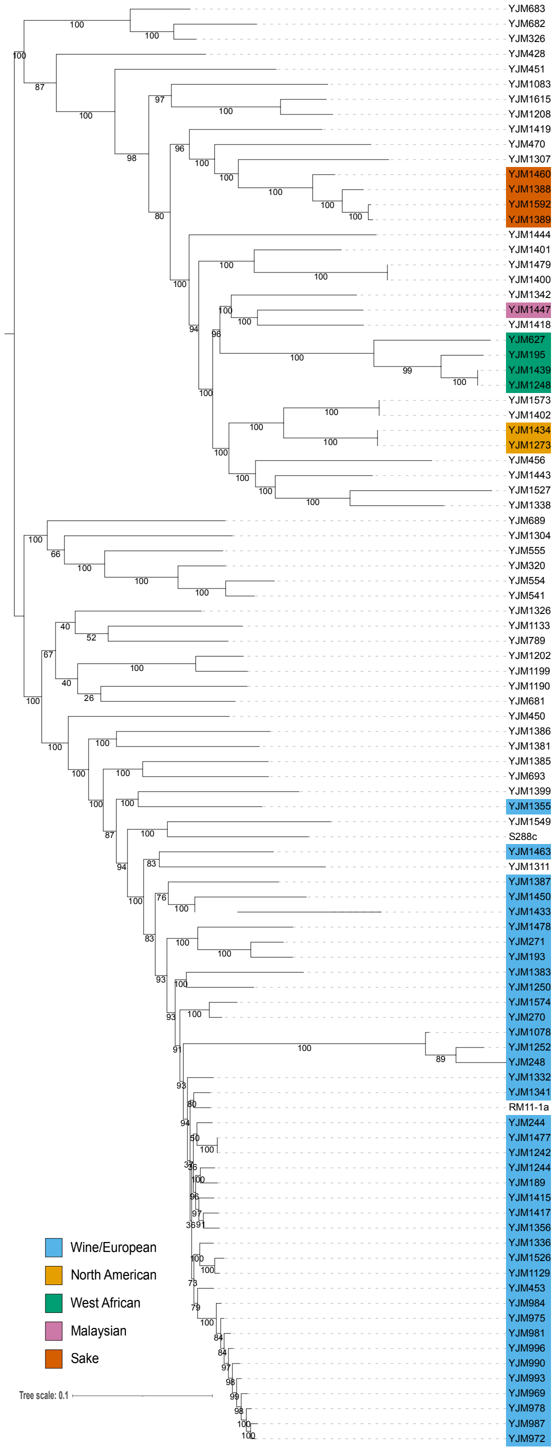

Figure C.3: Core SNP phylogeny of 96 *S. cerevisiae* strains. The phylogeny was inferred on 73,670 parsimony informative sites that were identified from 2,344 single-copy groups. Values on branches represent the bootstrap support obtained through 10,000 bootstrap replications. The root is placed at the midpoint of the tree.

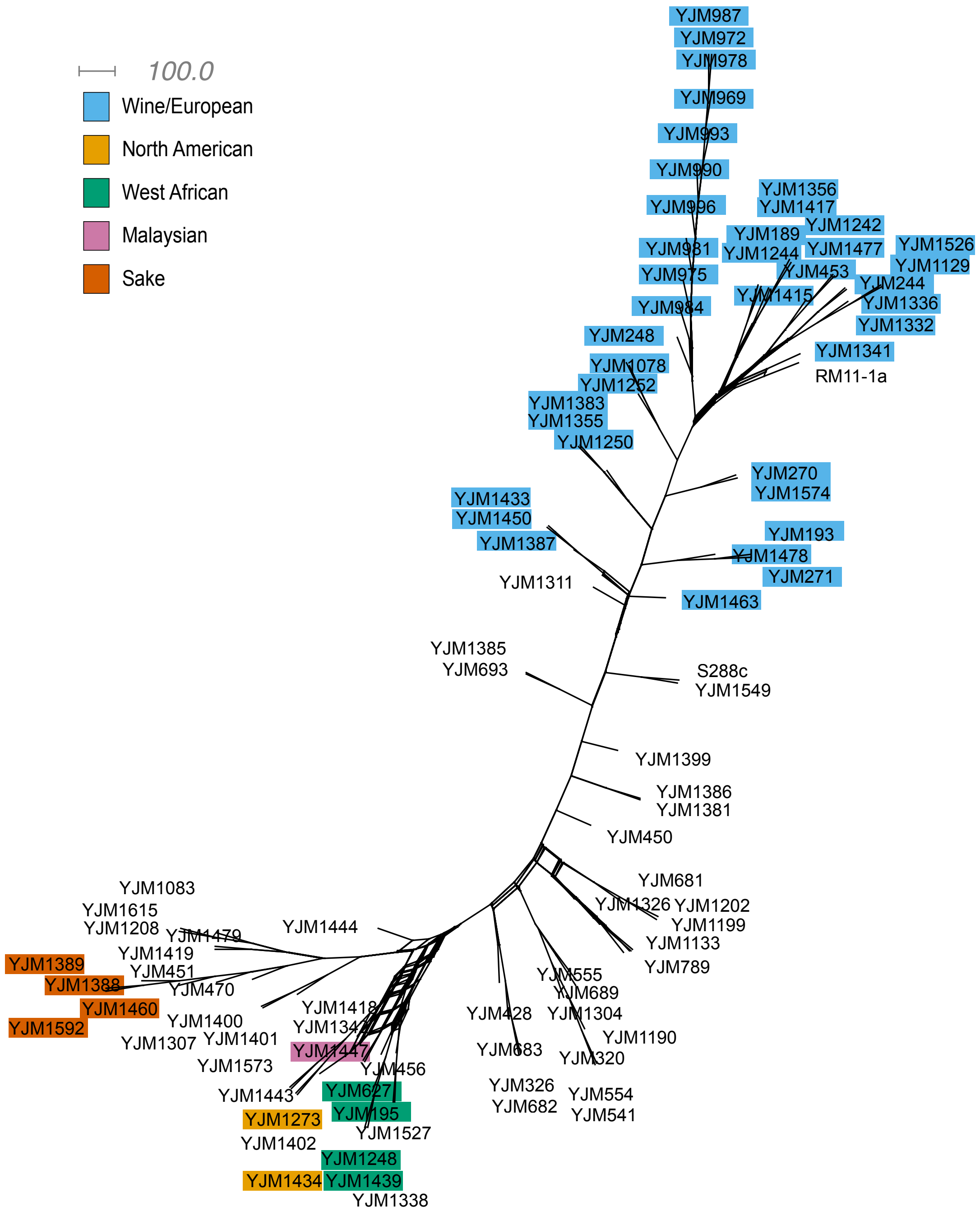

Figure C.4: Split network tree of 96 *S. cerevisiae* strains. This is a different representation of the core SNP phylogeny, with every supported split being visualized.

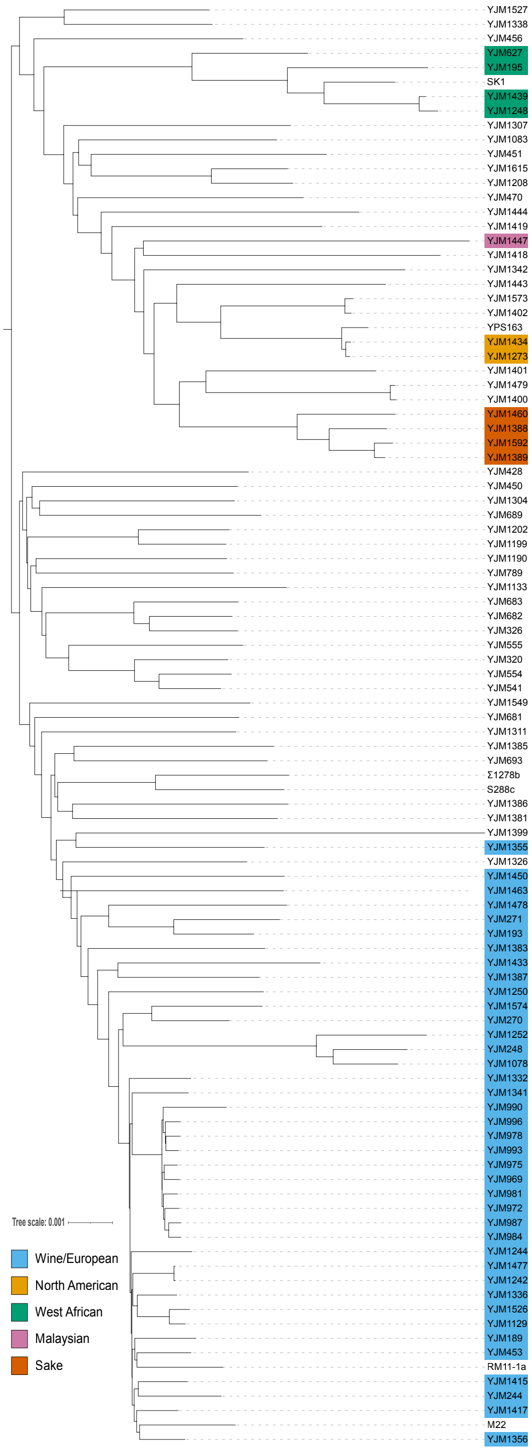

Figure C.5: k-mer distance tree of 100 *S. cerevisiae* strains. The phylogeny was inferred on pairwise distances calculated from 17.2 million k-mers. The root is placed at the midpoint of the tree.

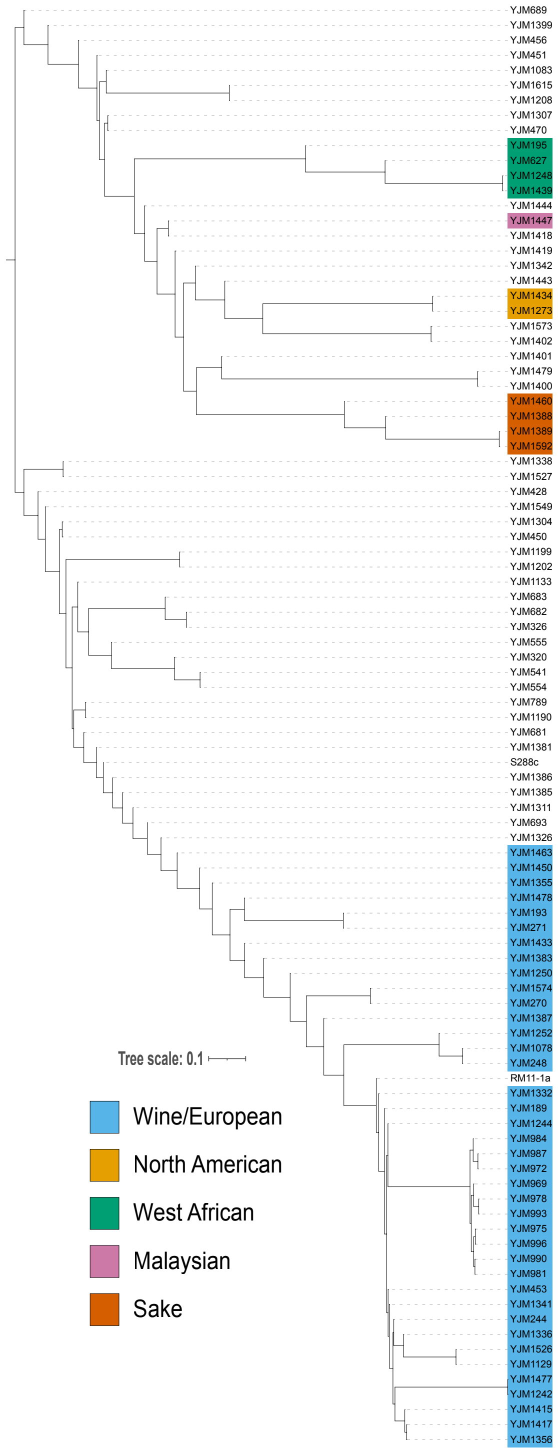

Figure C.6: Consensus tree of 96 *S. cerevisiae* strains. The phylogeny was inferred on 5,731 gene trees. The root is placed at the midpoint of the tree.

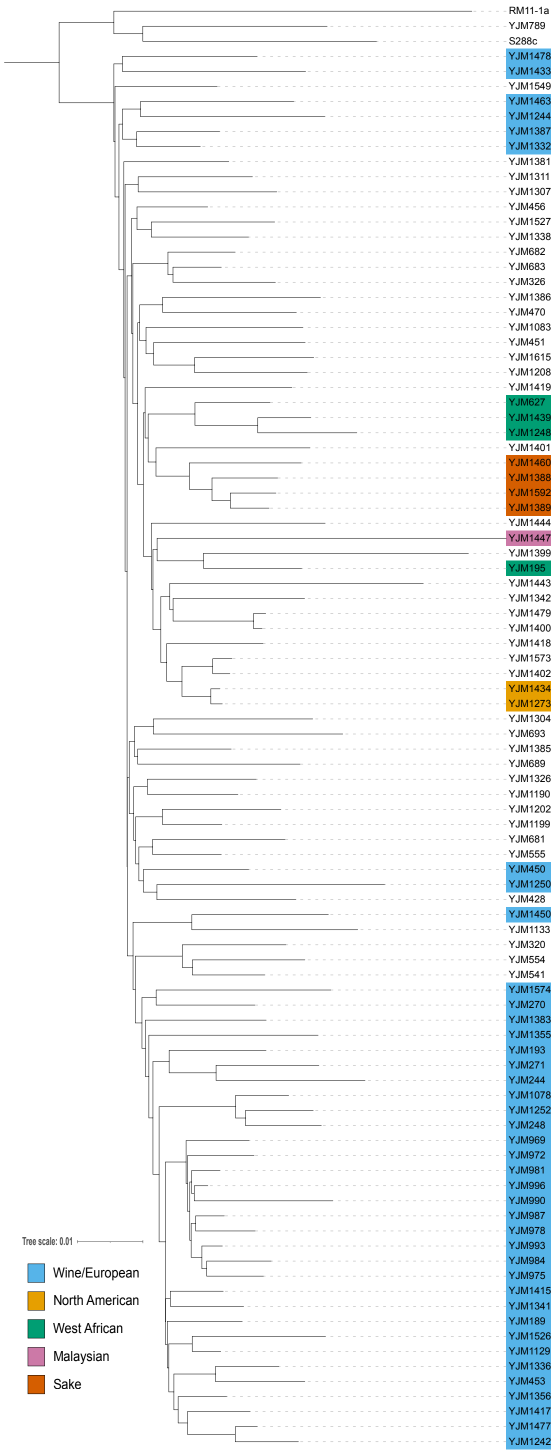

Figure C.7: Gene distance tree of 96 *S. cerevisiae* accessions. The phylogeny was inferred on pairwise distances calculated from counting shared genes in 5,731 homology groups. The root is placed at the midpoint of the tree.

D Appendix: Nextstrain SARS-CoV-2 phylogeny

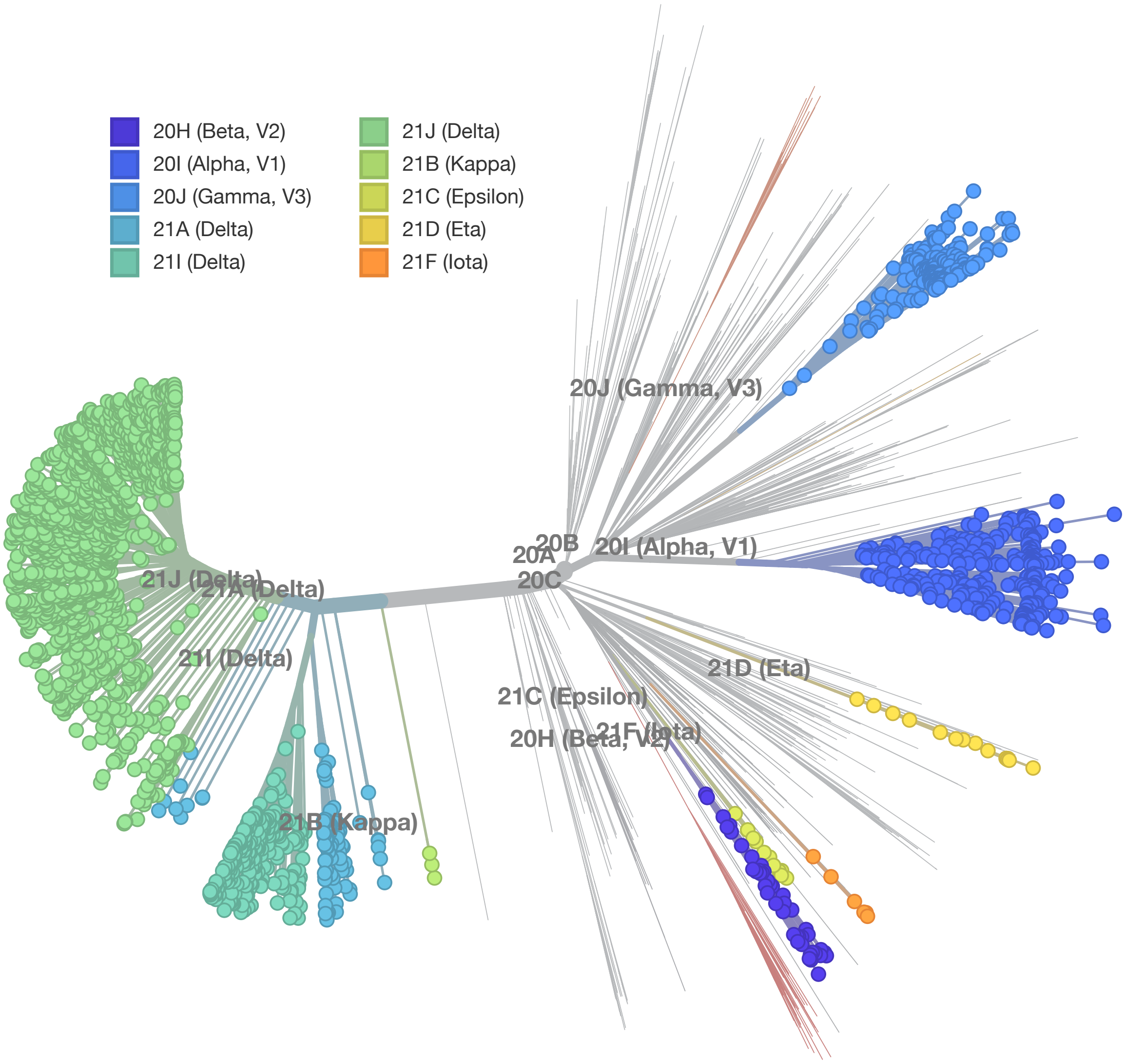

Figure D.1: Unrooted Nextstrain phylogeny of 3,544 SARS-CoV-2 genomes. Only variants of concern and variants of interest are highlighted. The tree was obtained on 1 November 2021.
